# Comparison of different predictive biomarker testing assays for PD-1/PD-L1 checkpoint inhibitors response: a systematic review and network meta-analysis

**DOI:** 10.3389/fimmu.2023.1265202

**Published:** 2023-09-26

**Authors:** Haotong Shi, Wenxia Zhang, Lin Zhang, Yawen Zheng, Taotao Dong

**Affiliations:** ^1^ Cheeloo College of Medicine, Shandong University, Jinan, China; ^2^ Department of Obstetrics and Gynecology, Qilu Hospital of Shandong University, Jinan, China

**Keywords:** anti-PD-1/PD-L1 inhibitors immunotherapy, biomarkers, predictive value of tests, solid tumor, meta-analysis

## Abstract

**Background:**

Accurate prediction of efficacy of programmed cell death 1 (PD-1)/programmed cell death ligand 1 (PD-L1) checkpoint inhibitors is of critical importance. To address this issue, a network meta-analysis (NMA) comparing existing common measurements for curative effect of PD-1/PD-L1 monotherapy was conducted.

**Methods:**

We searched PubMed, Embase, the Cochrane Library database, and relevant clinical trials to find out studies published before Feb 22, 2023 that use PD-L1 immunohistochemistry (IHC), tumor mutational burden (TMB), gene expression profiling (GEP), microsatellite instability (MSI), multiplex IHC/immunofluorescence (mIHC/IF), other immunohistochemistry and hematoxylin-eosin staining (other IHC&HE) and combined assays to determine objective response rates to anti–PD-1/PD-L1 monotherapy. Study-level data were extracted from the published studies. The primary goal of this study was to evaluate the predictive efficacy and rank these assays mainly by NMA, and the second objective was to compare them in subgroup analyses. Heterogeneity, quality assessment, and result validation were also conducted by meta-analysis.

**Findings:**

144 diagnostic index tests in 49 studies covering 5322 patients were eligible for inclusion. mIHC/IF exhibited highest sensitivity (0.76, 95% CI: 0.57-0.89), the second diagnostic odds ratio (DOR) (5.09, 95% CI: 1.35-13.90), and the second superiority index (2.86). MSI had highest specificity (0.90, 95% CI: 0.85-0.94), and DOR (6.79, 95% CI: 3.48-11.91), especially in gastrointestinal tumors. Subgroup analyses by tumor types found that mIHC/IF, and other IHC&HE demonstrated high predictive efficacy for non-small cell lung cancer (NSCLC), while PD-L1 IHC and MSI were highly efficacious in predicting the effectiveness in gastrointestinal tumors. When PD-L1 IHC was combined with TMB, the sensitivity (0.89, 95% CI: 0.82-0.94) was noticeably improved revealed by meta-analysis in all studies.

**Interpretation:**

Considering statistical results of NMA and clinical applicability, mIHC/IF appeared to have superior performance in predicting response to anti PD-1/PD-L1 therapy. Combined assays could further improve the predictive efficacy. Prospective clinical trials involving a wider range of tumor types are needed to establish a definitive gold standard in future.

## 1 Introduction

Since the approval of anti-PD-1/PD-L1 inhibitors in the treatment of melanoma in 2014, the overall survival of patients has improved significantly. However, anti-PD-1/PD-L1 immunotherapy still has many shortcomings, such as PD-1/L1-induced immune-related adverse events (irAEs) and hyperprogression (1). It is important to predict patients’ response to PD-1/PD-L1 immunotherapy based on the consideration of medical economics.

Various testing assays have been approved to predict the efficacy of anti-PD-1/PD-L1 immunotherapy response. Food and Drug Administration (FDA) has approved PD-1/PD-L1 IHC, TMB, proficient mismatch repair (pMMR) proteins, deficient mismatch repair (dMMR), and MSI-high (MSI-H) for specific tumor types and drugs as companion or complementary diagnostics (2). Similarly, European Communities (CE) and National Medical Products Administration (NMPA) have carried out their own standards on companion diagnostics and prediction assay applications.

PD-L1 IHC, the first approved companion diagnostic biomarker, aims to detect PD-1/PD-L1 expression on tumor cells or inflammatory cells. However, the efficacy of IHC may be influenced by the experience of pathologists, tumor types examined, and the used scoring methods. Researchers are now exploring the optimal detecting assay and scoring methods for specific tumors (3).

TMB has been found to increase neoantigens of major histocompatibility complexes (MHC) in various cancers, which leading to better immunotherapy response in patients. Increasing evidence indicates that different tumor types own various expression levels of TMB. TMB is usually assessed by next-generation sequencing (NGS) platforms, though standards of threshold and application methods need to be defined exactly to enhance accuracy across different tumor types. This would entail considerations such as genome coverage, workflow, and appropriate cutoff values (4). MSI and GEP display the difference in gene expression as well. MSI-H phenotype arises from numerous frameshift mutations due to deficits of the MMR system (5). Patients with MSI-H are more likely to suffer from various cancers, including colorectal cancer. MMR proteins, which could be detected by IHC, polymerase chain reaction (PCR), and gene sequencing, are now being used to identify MSI-H patients in various cancer types.

Detection and evaluation of tumor microenvironment (TME) have also been explored in recent years (6). For example, researchers have found that the epithelial-mesenchymal transition (EMT)- and stroma-related gene expression status is related to patients’ tumorigenesis and drug resistance (7, 8). mIHC/IF and gene sequencing technique could offer more chances to verify (9). GEP could also allow the integrations of different gene signatures and training models to predict prognosis and drug response based on the results of DNA-microarray and RNA sequencing (RNA-Seq) (10–12). Some researchers have also explored the combined approaches, such as TMB+GEP or TMB+IHC, since such predictors could work through different mechanisms or may be positively correlated with each other. All biomarker assays mentioned above present novel opportunities to predict the response rate of PD-1/PD-L1 inhibitors.

Assessment and evaluation of diagnostic tests could also benefit from the increasing diagnostic test accuracy (DTA) studies and the continuous development of statistical methods. In the era of evidence-based medicine, meta-analysis plays an important role in integrating of different studies with pairs of intervention using various methodological methods. To enable the comparison of different assays with limited data and generate a whole scale ranking results, NMA turned out to be a better tool to indirectly compare and jointly analyze three or more DTA studies simultaneously.

In this study, we compared the diagnostic accuracy of seven biomarker testing assays, including PD-L1 IHC, TMB, GEP, MSI, mIHC/IF, other IHC&HE, as well as combined assays for predicting anti-PD-1/PD-L1 immunotherapeutic response. Diagnostic accuracy measures used in this study included sensitivity, specificity, relative sensitivity, relative specificity, PPV, NPV, relative predictive values, DOR, and superiority index (13). It is believed that the NMA performed here could provide stronger clinical evidence for current medical practice.

## 2 Methods

This NMA was performed according to the Preferred Reporting Items for Systematic Reviews and Meta-analyses (PRISMA) NMA checklist.

### 2.1 Eligibility criteria

The included research articles in this study were based on real-world data, and English translations were available. The studies were required to conduct PD-1/PD-L1 monotherapies and utilize at least two predictive biomarker testing assays on pre-treatment tissue samples. These assays could include PD-L1 IHC, TMB, GEP, MSI, mIHC/IF, HE for tumor-infiltrating lymphocytes (TIL), or other IHC methods. Each biomarker testing assay should provide sufficient information to determine the objective response rate (ORR) or non-progression rate (NPR) and allow for the calculation of sensitivity and specificity. If any testing assay had fewer than 15 tissue samples, it would not be considered. Hematologic cancers and flow cytometry studies on tumor lysates were excluded.

### 2.2 Search strategy and data collection

We systematically searched PubMed, Embase, and the Cochrane Library database for relevant studies and their errata (till February 2023). Additionally, we manually searched articles related to relevant clinical trials. For example, the search formula of Embase included: (“Immunohistochemistry “ OR “ Tumor mutational burden “ OR “ gene expression profiling “ OR “ multiplex immunofluorescence “ OR “ neoantigen load “ OR “ Immunofluorescence “)[Find articles with these terms] AND (“Pembrolizumab “ OR “ Nivolumab “ OR “ Durvalumab “ OR “ Toripalimab “ OR “ Camrelizumab “ OR “ Atezolizumab “ OR “ Avelumab “ OR “ Avelumab “ OR “ Budigalimab “)[Title, abstract or author-specified keywords] AND (Research articles)[Filter]. The intact search formula and results were in the **Supplementary material**.

Necessary information from eligible studies was extracted by three researchers independently and all inconsistencies were settled by discussion. The trial name, first author, year of publication, sample size, trial phase, tumor type, PD-1/PD-L1 antibody, and index test assay was recorded. To calculate sensitivity and specificity for each index test, we organized ORR-related information into a 2x2 table. We used Youden’s index, which combines values for sensitivity and specificity to indicate test accuracy, to select the best-performing threshold among multiple thresholds. If a clinical trial has multiply publications, the one with most complete information was adopted.

### 2.3 Statistical analysis and quality assessment

The main outcomes were calculated by NMA. As for Bayesian NMA, the ANOVA model made it possible to use the original data and arm-based (AB) model (14). The latter shows superiority to contrast-based (CB) models by accommodating more complex variance-covariance structures. NMA was mainly performed with the R package “Rstan” (R version 4.2.2). In order to improve accuracy and compare diagnostic assays one by one, calculations were repeated 7 times (model_code = model, chains = 2, iterations = 10000, warmup = 5000, thin = 5), and then, we draw league tables for relative comparations. Given numerical variance, we chose the median of sensitivity, specificity, PPV, NPV, SROC, and superiority index.

The Midas module for DTA meta-analysis facilitated validation of results and assessment of heterogeneity by forest plot and I^2^ analysis for every 7 biomarker modalities. Sensitivity, specificity, DOR, and summary receiver operating characteristic (SROC) curves and their associated area under the curve (AUC) were analyzed by Midas, which employs a bivariate mixed-effects logistic regression modeling framework and empirical Bayesian predictions. Publication bias of studies was also evaluated by Deeks’ funnel plot asymmetry test (p<0.05 indicating significant asymmetry). The network graphs package on Stata were used to draw the network graphs. Meta-analysis and drawing figures were fulfilled in Stata (17.0 MP—Parallel Edition).

The QUADAS-C (Quality Assessment of Diagnostic Accuracy Study) tool was used to assess the risk of bias and applicability in each selected study. There were 4 sections for risk of bias: patient selection, index test, reference standard, and flow and timing; meanwhile, concerns regarding applicability were presented in 3 sections: patient selection, index test, and reference standard.

## 3 Results

### 3.1 Systematic review and characteristics of the included studies

**Figure 1:**
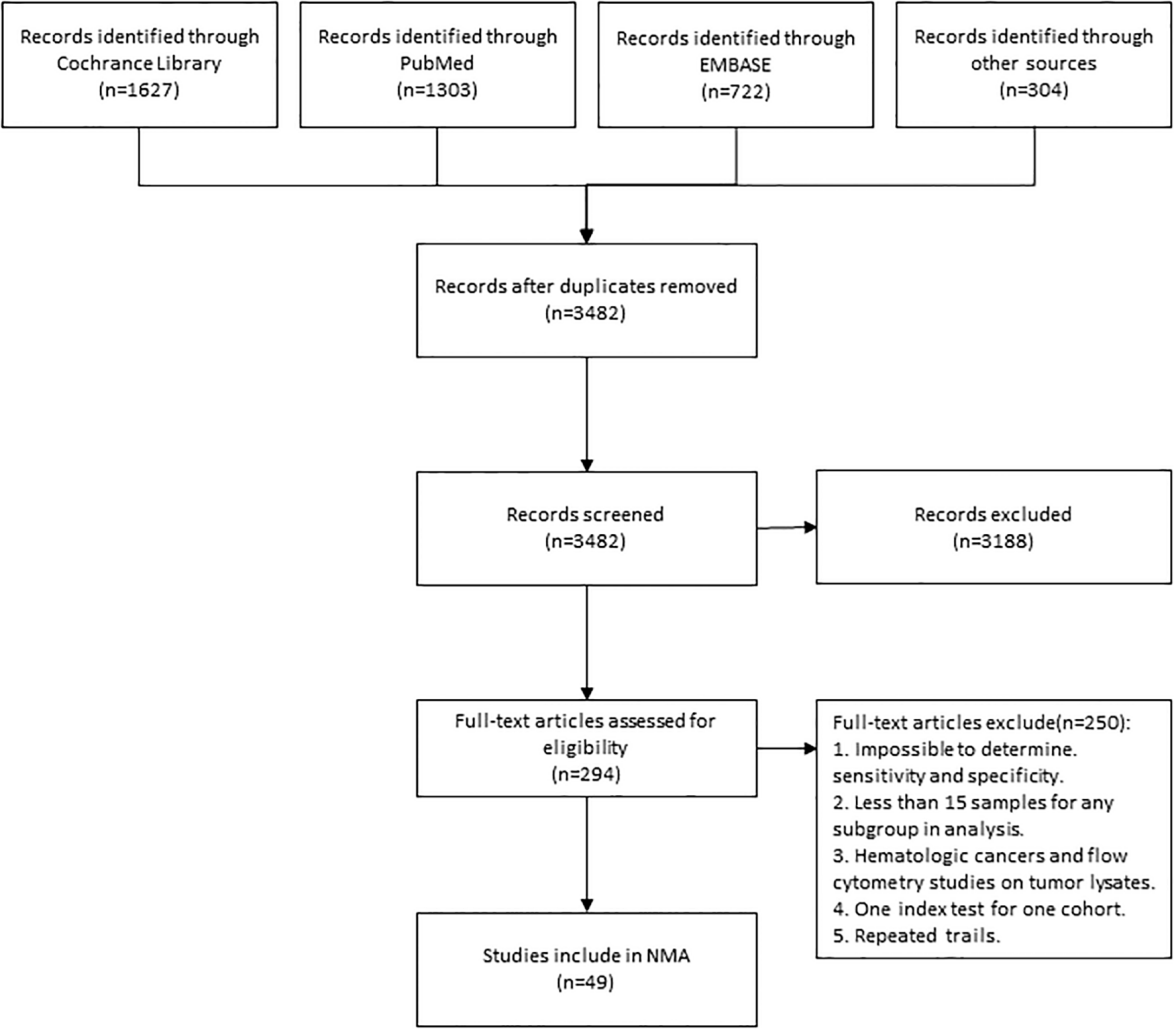
Flowchart Showing Literature Search and Study Selection. The study process followed the PRISMA guidelines. NMA, network meta-analysis.

**Figure 2:**
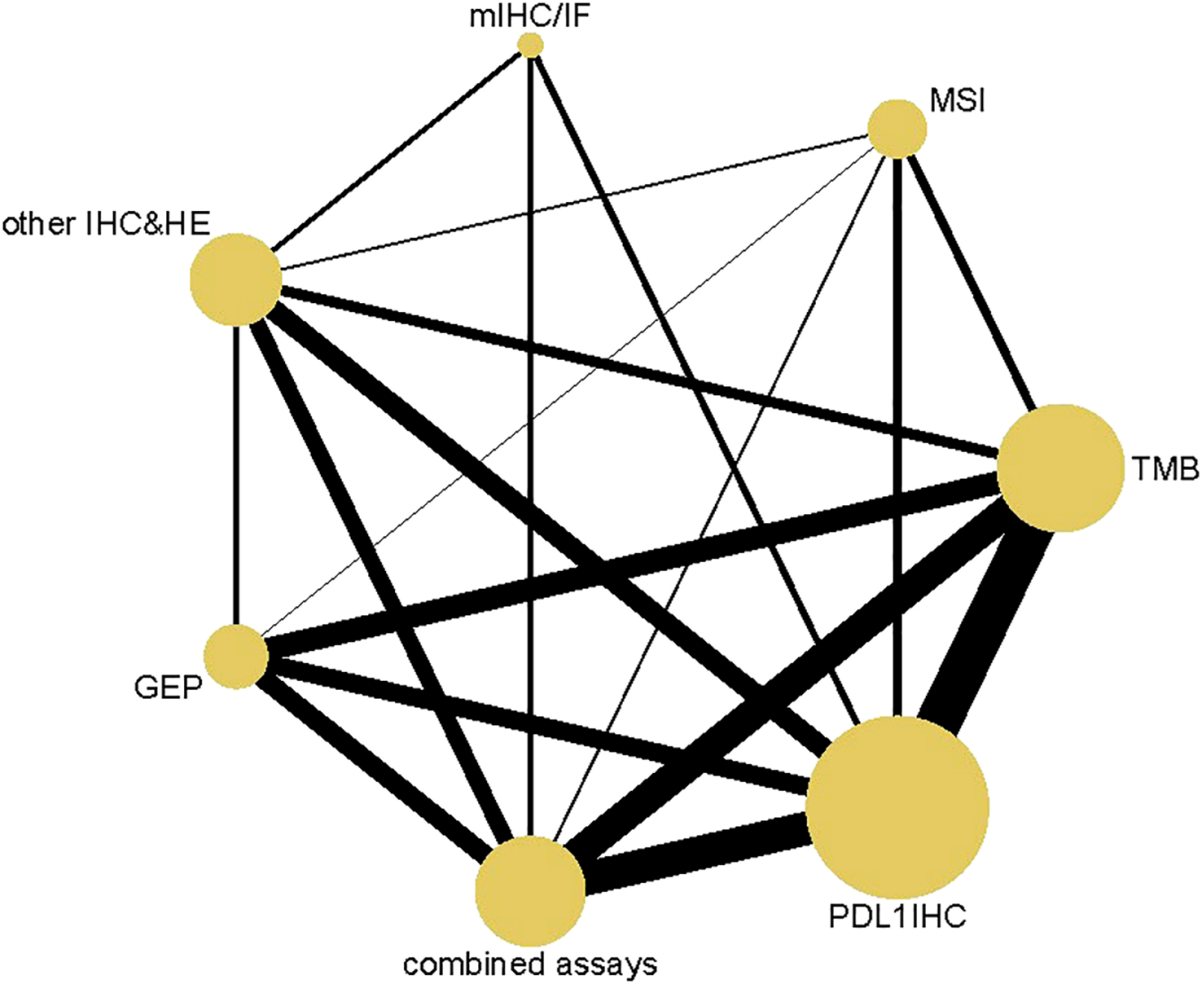
Network Plot. Both nodes and lines are weighted according to the number of studies involved in each treatment and direct comparison, respectively. PD-L1 IHC, Programmed cell death ligand 1 immunohistochemistry; TMB, Tumor mutational burden; GEP, Gene expression profiling; MSI, Microsatellite instability; mIHC/IF, Multiplex immunohistochemistry/immunofluorescence; other IHC&HE, Other Immunohistochemistry and hematoxylin-eosin staining.

3652 articles from databases and an additional 304 articles related to clinical trials were retrieved in total. After removing duplicates and glancing at the abstracts and titles, 294 articles were identified for full-text scrutiny. The literature search and study selection flow were recorded in **Figure 1**. Ultimately, a total of 49 studies involving 5322 patients were included in our analysis. 144 diagnostic index tests were extracted across all 49 studies, comprising PD-L1 IHC (n=46) (15–58), TMB (n=27) (15–33, 58–62), combined assays (n=22) (7, 16, 18, 20, 23, 31, 34–38, 61, 62), other IHC&HE (n=19) (7, 16–18, 21, 30, 33–35, 37–45), MSI (n=13) (21, 39, 46-53, 58, 61), GEP (n=13) (7, 16, 20, 23, 51, 53–56, 60, 62) and mIHC/IF (n=4)(36, 37, 43, 57). HE staining was used to score TIL. The situation where testing assays had been directly compared was represented by a network plot (**Figure 2**). 15 types of tumors accounted for the majority of the studies, while 7 studies (18, 20, 27, 31, 42, 60, 61) involved several solid tumors. 8 of 13 MSI tests (39, 46, 47, 50–53, 58) detected gastrointestinal cancer. The summary of included articles and details of studies can be found in **Supplementary Tables 1**, **2**.

### 3.2 Sensitivity, specificity, PPV and NPV

The sensitivity and specificity of NMA were summarized in **Table 1**. Among the diagnostic index tests, mIHC/IF (0.76, 95% CI: 0.57-0.89) exhibited the highest sensitivity, whereas GEP (0.52, 95% CI: 0.42-0.63), multi-assay (0.46, 95% CI: 0.39-0.52) and MSI (0.42, 95% CI: 0.30-0.53) have low efficacy. Other IHC&HE (0.66, 95% CI: 0.57-0.73), PD-L1 IHC (0.63, 95% CI: 0.59-0.67), and TMB (0.62, 95% CI: 0.56-0.68) presented similar sensitivities to rule out stable disease and progressive disease. As for specificity, MSI (0.90, 95% CI: 0.85-0.94) and combined assays (0.84, 95% CI: 0.79-0.87) performed better than the others. The specificities of the remaining testing assays were quite close, with TMB, other IHC&HE, PD-L1 IHC, GEP, and mIHC/IF having specificities of 0.65 (95% CI: 0.60-0.70), 0.63 (95% CI: 0.55-0.69), 0.61 (95% CI: 0.58-0.64), 0.61 (95% CI: 0.52-0.69) and 0.57 (95% CI: 0.39-0.73), respectively.

**Table 1:** Sensitivity, specificity, PPV, NPV, and diagnostic odds ratio (DOR) and superiority index by network meta-analysis.

Test	Ranks	Sensitivity	Ranks	Specificity	Ranks	PPV	Ranks	NPV	Ranks	DOR	Ranks	Superiority Index
**mIHC/IF**	1	0.76 (0.57,0.89)	7	0.57 (0.39,0.73)	6	0.33 (0.28,0.38)	1	0.86 (0.79,0.91)	2	5.09 (1.35,13.90)	2	2.86 (0.14,9.00)
**other IHC&HE**	2	0.66 (0.57,0.73)	4	0.63 (0.55,0.69)	4	0.36 (0.31,0.41)	2	0.85 (0.82,0.88)	4	3.22 (1.97,5.05)	3	2.66 (0.20,7.00)
**PD-L1 IHC**	3	0.63 (0.59,0.67)	5	0.61 (0.58,0.64)	5	0.34 (0.32,0.37)	4	0.84 (0.82,0.85)	6	2.67 (2.17,3.25)	5	1.15 (0.14,5.00)
**TMB**	4	0.62 (0.56,0.68)	3	0.65 (0.60,0.70)	3	0.38 (0.34,0.42)	3	0.84 (0.82,0.86)	5	3.10 (2.21,4.25)	1	2.94 (0.20,7.00)
**GEP**	5	0.52 (0.43,0.63)	6	0.61 (0.52,0.69)	7	0.33 (0.28,0.38)	7	0.80 (0.77,0.84)	7	1.81 (1.31,2.40)	7	0.33 (0.14,1.00)
**combined assays**	6	0.46 (0.39,0.54)	2	0.84 (0.79,0.87)	2	0.48 (0.43,0.53)	6	0.82 (0.80,0.84)	3	4.44 (3.19,5.93)	6	1.14 (0.33,3.00)
**MSI**	7	0.42 (0.30,0.53)	1	0.90 (0.85,0.94)	1	0.56 (0.45,0.67)	5	0.83 (0.79,0.86)	1	6.79 (3.48,11.91)	4	1.65 (1.00,5.00)

PPV, Positive predictive value; NPV, Negative predictive value; PD-L1 IHC, Programmed cell death ligand 1 immunohistochemistry; TMB, Tumor mutational burden; GEP, Gene expression profiling; MSI, Microsatellite instability; mIHC/IF, Multiplex immunohistochemistry/immunofluorescence; other IHC&HE, Other Immunohistochemistry and hematoxylin-eosin staining.

**Table 1** also revealed that the PPV for each assay was below 0.60, indicating that positive results may not correctly predict the response to PD-1/PD-L1 checkpoint inhibitors. MSI (0.56, 95% CI: 0.45-0.67) had the highest PPV, while GEP (0.33, 95% CI: 0.28-0.38) was the lowest. However, all assays provided relatively good performance in NPV, with even the lowest being near 0.80 (GEP: 0.8, 95% CI: 0.77-0.83). This suggested that these assays were useful in providing evidence to refuse immunologic therapy due to the accuracy of figuring out non-responsive patients.

### 3.3 Rankings, DOR and superiority index

Relative sensitivity, relative specificity, relative PPV, and relative NPV were shown in the league table (**Table 2**). From the league table for relative sensitivity (lower triangle of **Table 2 (A)**, we can see that mIHC/IF, other IHC&HE, and PD-L1 IHC had similar efficacy and performed better than TMB, GEP, combined assays, and MSI according to the relative risk (RR) values. The upper triangle of **Table 2(A)** represented the relative specificity, MSI and multi-assay showed superiority to the other, meanwhile, the remaining tests exhibited comparable efficacy. Similarly, MSI and combined assays demonstrated higher relative PPVs among assays, as shown in the lower triangle of **Table 2(B)**. There was no difference among relative NPVs (upper triangle of **Table 2(B)**.

**Table 2:** Relative sensitivity, relative specificity, relative PPV, and relative NPV by network meta-analysis.

(A)							
**mIHC/IF**	0.92 (0.69,1.21)	0.96 (0.83,1.17)		0.85 (0.62,1.19)	0.87 (0.60,1.15)	**0.67 (0.46,0.90)**	**0.63 (0.43,0.83)**
**RANK7**	**GEP**	1.03 (0.99,1.11)		1.00 (0.83,1.17)	0.94 (0.79,1.08)	**0.73 (0.62,0.83)**	**0.68 (0.57,0.78)**
	**RANK6**	**PD-L1 IHC**		0.99 (0.88,1.12)	0.94 (0.86,1.04)	**0.73 (0.68,0.79)**	**0.68 (0.63,0.73)**
**RANK1**		**RANK5**		**other IHC&HE**	0.95 (0.82,1.10)	**0.74 (0.65,0.84)**	**0.69 (0.60,0.78)**
**mIHC/IF**	**RANK2**			**RANK4**	**TMB**	**0.78 (0.71,0.85)**	**0.72 (0.65,0.79)**
0.90 (0.70,1.21)	**other IHC&HE**	**RANK3**			**RANK3**	**combined assays**	**0.93 (0.87,0.99)**
0.86 (0.69,1.14)	1.00 (0.85,1.10)	**PD-L1 IHC**		**RANK4**		**RANK2**	**MSI**
0.85 (0.66,1.15)	0.96 (0.81,1.11)	1.01 (0.95,1.12)		**TMB**	**RANK5**		**RANK1**
**0.72 (0.53,0.97)**	**0.78 (0.64,0.99)**	**0.79 (0.69,0.91)**		0.85 (0.68,1.04)	**GEP**	**RANK6**	
**0.63 (0.48,0.85)**	**0.73 (0.58,0.84)**	**0.70 (0.65,0.83)**		**0.74 (0.61,0.90)**	0.80 (0.67,1.08)	**combined assays**	**RANK7**
**0.57 (0.38,0.81)**	**0.63 (0.46,0.82)**	**0.74 (0.68,0.81)**		**0.67 (0.48,0.88)**	0.78 (0.56,1.05)	0.89 (0.61,1.21)	**MSI**
(B)							
**GEP**	0.99 (0.94,1.03)	0.97 (0.91,1.04)		0.96 (0.92,1.00)	0.96 (0.91,1.00)	**0.94 (0.89,0.99)**	**0.94 (0.87,1.03)**
**RANK7**	**combined assays**	0.99 (0.93,1.05)		0.98 (0.94,1.01)	0.97 (0.94,1.00)	**0.96 (0.92,10.0)**	**0.96 (0.90,1.04)**
	**RANK6**	**MSI**		0.99 (0.93,1.04)	0.98 (0.93,1.03)	**0.97 (0.91,1.03)**	**0.97 (0.89,1.06)**
**RANK1**		**RANK5**		**PD-L1 IHC**	1.00 (0.97,1.03)	**0.98 (0.94,1.02)**	**0.98 (0.92,1.07)**
**MSI**	**RANK2**			**RANK4**	**TMB**	**0.99 (0.94,1.03)**	**0.99 (0.92,1.08)**
0.86 (0.70,1.09)	**combined assays**	**RANK3**			**RANK3**	**other IHC&HE**	**1.00 (0.93,1.09)**
**0.68 (0.54,0.85)**	**0.79 (0.68,0.90)**	**TMB**		**RANK4**		**RANK2**	**mIHC/IF**
**0.64 (0.50,0.81)**	**0.74 (0.62,0.88)**	0.95 (0.78,1.12)		**other IHC&HE**	**RANK5**		**RANK1**
**0.61 (0.50,0.77)**	**0.72 (0.62,0.81)**	**0.91 (0.81,1.03)**		0.97 (0.82,1.14)	**PD-L1 IHC**	**RANK6**	
**0.60 (0.38,0.86)**	**0.70 (0.46,0.96)**	**0.89 (0.59,1.22)**		**0.94 (0.64,1.31)**	0.97 (0.65,1.32)	**mIHC/IF**	**RANK7**
**0.59 (0.46,0.76)**	**0.69 (0.58,0.81)**	**0.88 (0.74,1.05)**		**0.94 (0.75,1.15)**	0.96 (0.80,1.13)	1.02 (0.69,1.50)	**GEP**

(A) Relative risk (RR) values and 95% CIs for sensitivity (lower triangle) and specificity (upper triangle) were in **Table 2**.

(B) Relative risk (RR) values and 95% CIs for PPV (lower triangle) and NPV (upper triangle) were in **Table 2**.

The values highlighted in bold indicated a significant difference between the two compared assays. Relative risk (RR) values <1.00 provided better predictive efficacy.

PD-L1 IHC, Programmed cell death ligand 1 immunohistochemistry; TMB, Tumor mutational burden; GEP, Gene expression profiling; MSI, Microsatellite instability; mIHC/IF, Multiplex immunohistochemistry/immunofluorescence; other IHC&HE, Other Immunohistochemistry and hematoxylin-eosin staining.

**Table 1** presented the odds of responsive patients in test positives versus the odds of responsive patients in test negatives as measured by the DOR. MSI (6.79, 95% CI: 3.48-11.91) has the highest DOR as its high specificity, followed by mIHC/IF (4.44, 95% CI: 3.19-5.93), largely driven by its high sensitivity. In contrast, the DOR for gene expression profiling (GEP) was noticeably lower at 1.81 (95% CI: 1.31-2.40). The high superiority index indicated biomarkers modality performs comparatively well in both sensitivity and specificity. In contrast, the low superiority index represents biomarkers that had a poor performance of at least one assessment measure. As **Table 1** summarized, the ranks of superiority index from highest to lowest were TMB, mIHC/IF, other IHC&HE, MSI, PD-L1 IHC, combined assays, and GEP.

### 3.4 Heterogeneity and quality assessment

**Figure 3:**
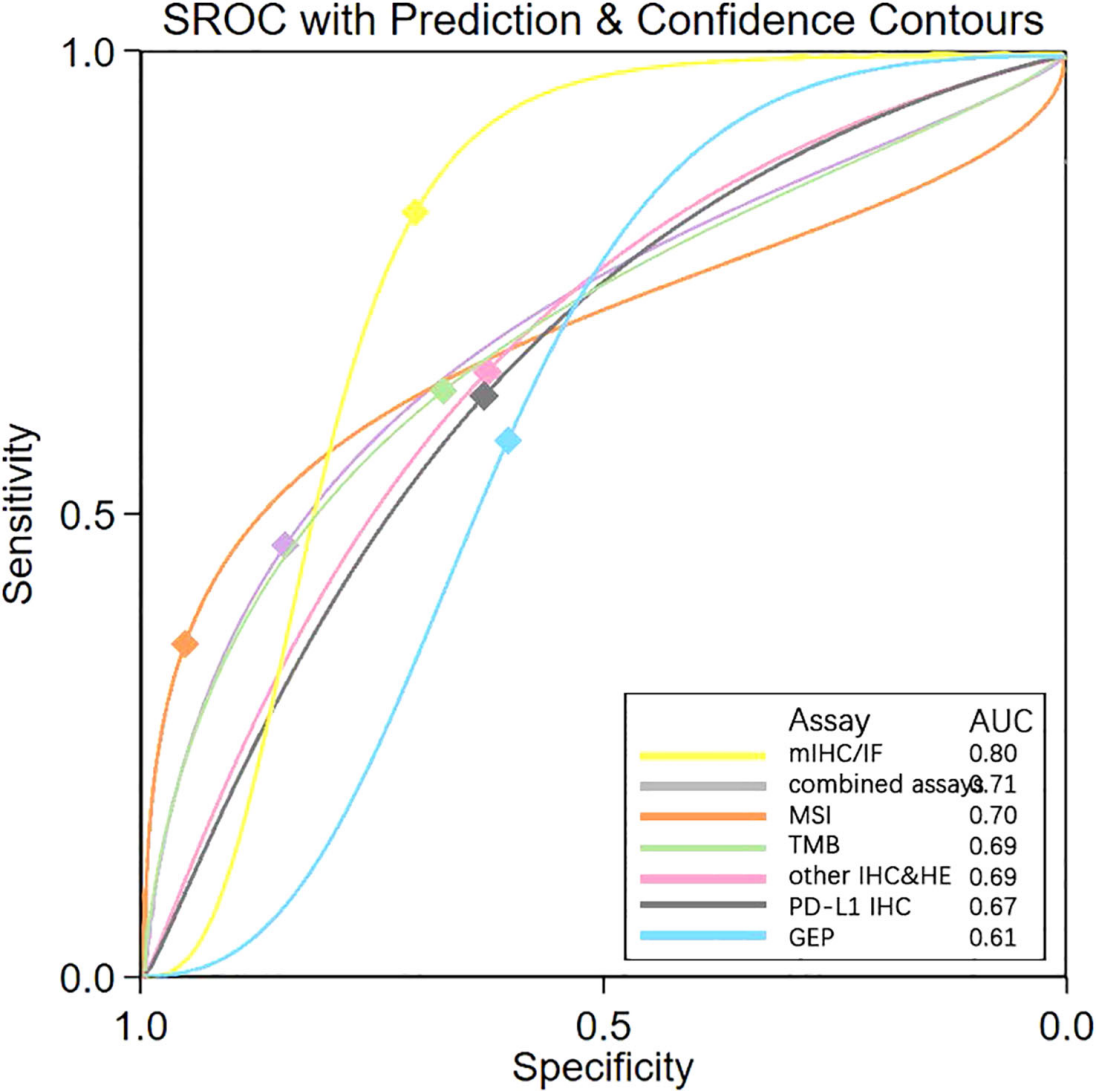
SROC Plot of “mIHC/IF” “combined assays” “MSI “ “TMB” “other IHC&HE” “PDL1 IHC” and “GEP” by Meta-analysis. SROC, Summary receiver operating characteristic curves; AUC, Area under the curve; PD-L1 IHC, Programmed cell death ligand 1 immunohistochemistry; TMB, Tumor mutational burden; GEP, Gene expression profiling; MSI, Microsatellite instability; mIHC/IF, Multiplex immunohistochemistry/immunofluorescence; other IHC&HE, Other Immunohistochemistry and hematoxylin-eosin staining.

To further validate these present results, a meta-analysis was conducted and revealed the same ranks of sensitivity, specificity, and DOR as NMA (**Table 3**). The value of sensitivity and specificity were very similar, indicating reliable results from the ANOVA model used in the NMA. SROC generated through meta-analysis displayed the AUC for each biomarker testing assay. mIHC/IF had the largest AUC (0.80), while GEP exhibited the smallest (0.61) and AUC of all others were close to 0.70 (**Figure 3**). Ranking trends for AUC and DOR were similar, indicating the reliability of our ranking results for NMA.

**Table 3:** Result validation by meta-analysis.

Ranks	Test	Sensitivity	Test	Specificity	Test	DOR
**1**	mIHC/IF	0.83 (0.14-0.99)	MSI	0.96 (0.88-0.99)	MSI	13 (6-9)
**2**	other IHC&HE	0.66 (0.55-0.75)	combined assays	0.85 (0.79-0.89)	mIHC/IF	12 (1-243)
**3**	PD-L1 IHC	0.63 (0.55,0.70)	TMB	0.68 (0.60-0.74)	multi-assay	5 (4-7)
**4**	TMB	0.63 (0.56-0.70)	other IHC&HE	0.63 (0.57-0.69)	other IHC&HE	3 (2-5)
**5**	GEP	0.58 (0.38-0.76)	PD-L1 IHC	0.63 (0.57.0.69)	TMB	4 (3,5)
**6**	combined assays	0.47 (0.39-0.55)	GEP	0.61 (0.51-0.69)	PD-L1 IHC	3 (2,4)
**7**	MSI	0.36 (0.23-0.52)	mIHC/IF	0.71 (0.45-0.88)	GEP	2 (1,4)

DOR, Diagnostic odds ratio; PD-L1 IHC, Programmed cell death ligand 1 immunohistochemistry; TMB, Tumor mutational burden; GEP, Gene expression profiling; MSI, Microsatellite instability; mIHC/IF, Multiplex immunohistochemistry/immunofluorescence; other IHC&HE, Other Immunohistochemistry and hematoxylin-eosin staining.

However, the heterogeneity for each biomarker was high due to the absence of testing standards and various tumor types and thresholds. Although we chose the best performance threshold, I^2^ was higher than 50% (**Supplementary Figure 1**). Nonetheless, publication bias wasn’t obvious (p>0.1), according to **Supplementary Figure 2**. QUADAS-C tools allowed us to evaluate the quality (**Supplementary Table 3**).

### 3.5 Subgroup analysis

We conducted NMA for two subgroups of studies: 10 studies focused on non-small cell lung cancer (NSCLC) (7, 23, 32–34, 45, 54, 58) and 12 studies centered around gastrointestinal tumors (19, 33, 39, 46, 47, 50–53, 58, 59) as reported in **Table 4** and **Table 5**. For NSCLC, mIHC/IF and multi-assay had high sensitivity (0.90, 95% CI: 0.44-1.00) and specificity (0.90, 95% CI: 0.84-0.95) separately. mIHC/IF, with only one study available, exhibited both high sensitivity and specificity (0.89, 95% CI: 0.69-0.98), suggesting its potential as a reliable biomarker modality. Further analysis based on the ranks of DOR and superiority index suggested mIHC/IF, multi-assay and other IHC&HE were better among the 6 testing assays investigated.

**Table 4:** Subgroup analysis of NSCLC by network meta-analysis.

Rank	Test	Sensitivity	Rank	Test	Relative Sensitivity	Rank	Test	DOR
**1**	mIHC/IF	0.90 (0.44,1.00)	**1**	mIHC/IF	1.42 (0.68,1.74)	**1**	mIHC/IF	1607584.12 (5.95,833493.27)
**2**	PD-L1 IHC	0.64 (0.56,0.72)	**2**	PD-L1 IHC	1.00 (1.00,1.00)	**2**	combined assays	6.55 (2.96,12.88)
**3**	TMB	0.59 (0.48,0.69)	**3**	TMB	0.92 (0.73,1.11)	**3**	other IHC&HE	6.20 (2.67,12.45)
**4**	other IHC&HE	0.55 (0.42,0.69)	**4**	other IHC&HE	0.87 (0.63,1.11)	**4**	PD-L1 IHC	3.30 (2.10,4.96)
**5**	GEP	0.44 (0.31,0.56)	**5**	GEP	0.68 (0.48,0.89)	**5**	TMB	2.88 (1.57,5.15)
**6**	combined assays	0.39 (0.27,0.50)	**6**	combined assays	0.61 (0.43,0.80)	**6**	GEP	1.68 (0.79,3.13)
Rank	Test	Specificity	Rank	Test	Relative Specificity	Rank	Test	Superiority Index
**1**	combined assays	0.90 (0.84,0.95)	**1**	combined assays	1.41 (1.25,1.59)	**1**	mIHC/IF	9.02 (1.00,11.00)
**2**	mIHC/IF	0.89 (0.69,0.98)	**2**	mIHC/IF	1.38 (1.06,1.61)	**2**	other IHC&HE	1.90 (0.33,7.00)
**3**	other IHC&HE	0.82 (0.71,0.89)	**3**	other IHC&HE	1.27 (1.08,1.47)	**3**	combined assays	1.07 (0.20,3.00)
**4**	GEP	0.67 (0.55,0.78)	**4**	GEP	1.04 (0.84,1.25)	**4**	PD-L1 IHC	0.83 (0.20,3.00)
**5**	TMB	0.66 (0.55,0.75)	**5**	TMB	1.02 (0.84,1.22)	**5**	TMB	0.65 (0.14,3.00)
**6**	PD-L1 IHC	0.64 (0.58,0.70)	**6**	PD-L1 IHC	1.00 (1.00,1.00)	**6**	GEP	0.18 (0.09,0.33)

DOR, Diagnostic odds ratio; PD-L1 IHC, Programmed cell death ligand 1 immunohistochemistry; TMB, Tumor mutational burden; GEP, Gene expression profiling; MSI, Microsatellite instability; mIHC/IF, Multiplex immunohistochemistry/immunofluorescence; other IHC&HE, Other Immunohistochemistry and hematoxylin-eosin staining.

**Table 5:** Subgroup analysis of gastrointestinal tumors by network meta-analysis.

Rank	Test	Sensitivity	Rank	Test	Relative Sensitivity	Rank	Test	DOR
**1**	other IHC&HE	0.72 (0.35,0.95)	**1**	other IHC&HE	1.00 (1.00,1.00)	**1**	other IHC&HE	7.24 (0.35,37.15)
**2**	PD-L1 IHC	0.56 (0.44,0.68)	**2**	PD-L1 IHC	0.84 (0.52,1.61)	**2**	MSI	5.73 (2.49,10.59)
**3**	TMB	0.55 (0.33,0.76)	**3**	TMB	0.82 (0.41,1.64)	**3**	PD-L1 IHC	2.73 (1.45,4.76)
**4**	MSI	0.40 (0.27,0.54)	**4**	MSI	0.60 (0.33,1.20)	**4**	mIHC/IF	1.92 (0.03,11.96)
**5**	mIHC/IF	0.37 (0.04,0.84)	**5**	mIHC/IF	0.55 (0.06,1.48)	**5**	TMB	1.62 (0.39,4.56)
**6**	GEP	0.06 (0.00,0.39)	**6**	GEP	0.10 (0.00,0.64)	**6**	GEP	0.45 (0,30,0.86)
Rank	Test	Specificity	Rank	Test	Relative Sensitivity	Rank	Test	Superiority Index
**1**	MSI	0.89 (0.82,0.92)	**1**	MSI	1.91 (1.09,4.19)	**1**	MSI	4.17 (1.00,7.00)
**2**	GEP	0.70 (0.28,0.96)	**2**	GEP	1.50 (0.49,3.52)	**2**	PD-L1 IHC	3.44 (0.33,7.00)
**3**	PD-L1 IHC	0.67 (0.60,0.73)	**3**	PD-L1 IHC	1.44 (0.81,3.14)	**3**	other IHC&HE	3.09 (0.14,9.00)
**4**	mIHC/IF	0.56 (0.17,0.91)	**4**	mIHC/IF	1.19 (0.32,2.88)	**4**	TMB	1.30 (0.14,7.00)
**5**	TMB	0.52 (0.32,0.71)	**5**	TMB	1.13 (0.50,2.65)	**5**	mIHC/IF	1.17 (0.11,7.00)
**6**	other IHC&HE	0.52 (0.21,0.82)	**6**	other IHC&HE	1.00 (1.00,1.00)	**6**	GEP	0.47 (0.09,3.00)

DOR, Diagnostic odds ratio; PD-L1 IHC, Programmed cell death ligand 1 immunohistochemistry; TMB, Tumor mutational burden; GEP, Gene expression profiling; MSI, Microsatellite instability; mIHC/IF, Multiplex immunohistochemistry/immunofluorescence; other IHC&HE, Other Immunohistochemistry and hematoxylin-eosin staining.

In the case of gastrointestinal cancers, MSI had high specificity (0.89, 95% CI: 0.82-0.92) and low sensitivity (0.40, 95% CI: 0.27-0.54). PD-L1 IHC along with other IHC&HE demonstrated relatively high DOR and superiority index, besides MSI.

Concerning that the majority of combined assays contained 3 models, namely, TMB+GEP (n=6) (16, 20, 23), TMB+PD-L1 IHC (n=6) (18, 20, 30), and PD-L1 IHC+other IHC&HE (n=5) (34–38). A meta-analysis was performed to explore sensitivity, specificity, DOR, and AUC (**Supplementary Figure 3**) in these models. TMB+PD-L1 IHC showed the best balance between sensitivity (0.89, 95% CI: 0.82-0.94) and specificity (0.68, 95% CI: 0.53-0.81) with high DOR (18, 95% CI: 9-37) and AUC (0.87, 95% CI: 0.84-0.90). Conversely, the other models yielded higher sensitivity but lower specificity compared to a single assay in the meta-analysis (**Supplementary Figure 3**).

## 4 Discussion

In this article, we compared 7 common biomarker testing assays to assess their efficacy in predicting response to PD-1/PD-L1 checkpoint inhibitors. mIHC/IF had the highest sensitivity (0.76, 95% CI: 0.57-0.89) and AUC (0.80), the second highest DOR (5.09, 95% CI: 1.35-13.90) and superiority index (2.86), but relative lower specificity (0.57, 95% CI: 0.39-0.73). Although MSI exhibited the highest DOR (6.79, 95% CI: 3.48-11.91), its application is mainly limited to gastrointestinal tumors. Despite being the most commonly used method in clinical practice, PD-L1 IHC had not demonstrated obvious advantages in terms of sensitivity, specificity, DOR, as well as superiority index. Yet, when PD-L1 IHC is combined with TMB, a notable increase in sensitivity (0.89, 95% CI: 0.82-0.94) was observed.

Our conclusion is in alignment with those from a previous meta-analyses that had addressed similar topics (63, 64), which indicated that mIHC/IF was superior to PD-L1 IHC, TMB and GEP in predicting response to PD-1/PD-L1 checkpoint inhibitors and that combinatorial assays could improve predictive efficacy. Yet, to our best of knowledge, our study was the first to use NMA to demonstrate the objective benefits of mIHC/IF in predicting patients’ response to PD-1/PD-L1 checkpoint inhibitors. Upon stratifying by tumor types, we also observed that mIHC/IF had both remarkable sensitivity and specificity in NSCLC. PD-L1, mIHC/IF and IHC also manifested relatively high DOR and superiority index in gastrointestinal cancers, which further substantiated the strengths of mIHC/IF.

To address the challenge of ranking multiple diagnostic tests simultaneously, statistical scientists have developed several new models based on the Bayesian setting for NMA of DTA studies (65), since traditional meta-analysis and NMA of intervention were not efficient enough to handle this issue. Multivariate extensions of meta-analysis models of DTA had been applied to NMA. In addition, the ANOVA model used in this NMA could facilitate ORR to be compared indirectly and rank testing assays directly (14). Researchers could also compare multiple thresholds per testing assay using certain models (66).

High sensitivity, DOR, and AUC of mIHC/IF collectively indicated its superiority in identification of potential patients who may benefit most from immunotherapy. mIHC/IF facilitates the acquisition of quantitative multiplexed data, which plays a pivotal role in deciphering the intricate relationship between tumor cells, their microenvironment, and antigen expressions at the single-cell level. This capability assumes paramount importance in understanding tumorigenesis, cancer progression, and immunotherapy responses. In all instances of mIHC/IF index testing, CD8 was included, and T cell antigen expression was examined. Various studies have established a link between T cells’ cytotoxicity and pro-inflammatory activity with patients prognosis through its regulation of inherent immunological function by tumor antigens like CD8 or PD-1 (67–70), which further supports the potency of antigens on tumor-infiltrating lymphocytes (TILs). However, false negative results obtained from mIHC/IF screening may exclude some patients who may could benefit from immunotherapy, suggesting the need to explore additional proteins and combined assays to improve specificity. To enhance the precision in scoring staining, many researchers have incorporated artificial intelligence with mIHC/IF, rendering it a relatively convenient and cost-effective method when compared to combined assays (71). Thus, our study has concluded that mIHC/IF had the best performance and a broad range of applications.

PD-L1 IHC, the most widely used assay, exhibited suboptimal performance in sensitivity, specificity, and DOR. As previously mentioned, TME is excessively intricate and heterogeneous to be comprehensively elucidated by a singular mechanism. Furthermore, expressions of PD-1 and PD-L1 exhibit considerable interpatient variability. These two factors collectively contribute to the suboptimal performance of PD-L1 IHC as a predictive marker. The possible reasons for such unsatisfactory results varied, including the lack of experience for pathologists, sample type examined, and IHC assays used (72). A meta-analysis that scrutinized and compared different IHC assays using tumor proportion score (TPS) revealed that the sensitivity and specificity values were similar except SP142 with lower sensitivity (73). The quantification and assessment of PD-1 protein expression through scoring methods varied among different assays, such as TPS, combined positivity score (CPS), and immune cell (IC) score (3). Gastrointestinal tumors were characterized by their most extensive proportions of MSI-H/dMMR, therefore, MSI status detection could be a reasonable approach to predict the response to immunotherapy. Subgroup analysis of gastrointestinal tumors indicated that MSI detection offered a valuable method for ruling out non-responsive patients due to its high specificity performance. MSI detection was also conducted in other solid tumors, including endometrial cancer, adrenocortical carcinomas, and multiple endocrine neoplasias (MENs). High specificity, DOR, and AUC of MSI suggested its potential applications in some other tumor types. Regrettably, generalization of MSI detections to a wider range of tumors may be prevented by the fact that most tumors in fact exhibit microsatellite stability (MSS) status.

Our efficacy rankings placed TMB and other IHC&HE in the middle, while GEP was ranked last, although they are closely related to crucial aspects of tumor immunology such as neoantigen, TME, and inflammatory gene signature. Nevertheless, it is important to note that the MSI status, TMB, and GEP serve as indicators of the gene phenotype, which is not directly associated with the primary mechanism of PD-1/PD-L1 immunotherapy compared to protein expression. The measurements obtained through MSI, TMB, and GEP reflect events upstream of gene expression, which may potentially diminish their predictive efficacy. Uncovering specific and precise gene pathways solely through these indicators can prove to be challenging. Whereas thresholds for TMB and GEP were mainly determined by proportions, other IHC&HE methods typically detected CD8 and TILs with different methods. This highlights the potential impossibility that some immature tests could have covered all types of tumors.

Combined assays provided more chances to improve the prediction accuracy in current challenging scenario. When TMB was combined with PD-L1 IHC, the performance of sensitivity was improved noticeably without sacrificing specificity. Ricciuti, B. et al. have explored the association of high TMB with other biomarkers and found that high TMB was related to higher proportions of tumor-infiltrating CD8+, PD1+ T cells, and high PD-L1 expression in cancer cells (74). Fumet, J.-D. et al. reported that tumors displaying high PD-L1/low CD8 TILs developed microenvironments conducive to tumor proliferation and exhibited poor outcomes (75). This may explain the enhanced efficacy of combined assays. Yet, the shortcomings of combined assays were high cost and technical complexity.

Despite nearly a decade of research on companion or complementary diagnostics for prediction purposes, the most effective indicators for PD-1/PD-L1 inhibitors have not yet been established for most tumors. While some testing assays such as mIHC/IF and combined tests hold potential values, there was still no perfect test with satisfactory sensitivity and specificity simultaneously in our analysis. Consequently, clinicians should exert appropriate caution when detecting predictive biomarkers and interpreting associated results. Additionally, it is believed that our NMA could provide supporting evidence to researchers and clinicians for amelioration of predictive tests in future.

## 5 Limitations

It is crucial to note that a high ORR doesn’t necessarily translate into a high OS. It is essential to take care when interpreting results based on studies that relied solely on ORR which may not take into account of OS or progressive rate. To mitigate bias, it is worth noting that the threshold we chose with Youden’s index may favor higher sensitivity and specificity. An article with two or more biomarker tests was selected, which may cause bias by giving up some robust data in each test. Moreover, there was a significant disparity between the number of studies conducted in PD-L1 IHC versus mIHC/IF. Last but not least, although our study mainly covered 15 types of tumors, the generalization of the conclusion still requires deliberation.

## 6 Conclusion

Various large prospective and retrospective studies have investigated biomarkers for the prediction of PD-1/PD-L1 checkpoint inhibitors response. According to our network meta-analysis, mIHC/IF had the best performance and a large range of applications. Given the diverse employment of mIHC/IF with different biomarkers across various studies, further investigations involving precise combinations are warranted to enhance prognostic prediction. When considering the selection of specific markers, it is crucial to take into account not only their efficiency and cost-effectiveness but also rely on substantiation from evidence derived from molecular mechanisms. Further exploration was required in combined assays of the high efficacy of TMB+PD-L1 IHC. Currently, there is a lack of studies or consensus regarding the workflow of companion or complementary diagnostics in this context. The existing approach is primarily based on clinicians’ acknowledgment, and we anticipate that future research will provide more foundational evidence to support these practices. What’ more, more evidence based medicine are needed to determine detailed testing modalities and thresholds for all types of tumors, e.g. advanced ovarian cancer. Clinicians should be cautious that the prognostic accuracy of each index test should be interpreted in a particular situation.

## Data availability statement

The original contributions presented in the study are included in the article/**Supplementary Material**. Further inquiries can be directed to the corresponding author.

## Author contributions

HS: Conceptualization, Data curation, Formal Analysis, Investigation, Writing – original draft. WZ: Investigation, Validation, Visualization, Writing – review & editing. LZ: Investigation, Validation, Visualization, Writing – review & editing. YZ: Conceptualization, Data curation, Formal Analysis, Supervision, Writing – review & editing. TD: Conceptualization, Funding acquisition, Software, Supervision, Writing – review & editing.
