# Supplementary material for: Comparison of different predictive biomarker testing assays for PD-1/PD-L1 checkpoint inhibitors response: a systematic review and network meta-analysis
Source: Front Immunol. 2023 Sep 26;14:1265202. doi: 10.3389/fimmu.2023.1265202 (PMC10562577; doi:10.3389/fimmu.2023.1265202)
Supplement: Supplementary file 1 [file DataSheet_1.docx]

Supplementary Material

Comparison of different predictive biomarker testing assays for PD-1/PD-L1 checkpoint inhibitors response: A systematic review and network meta-analysis

Haotong Shi, Yawen Zheng*, Taotao Dong*

*** Correspondence:** Taotao Dong: dongtt82@163.com, Yawen Zheng, zhengyawen1993@gmail.com

# Supplementary Data

Eq 1 Embase Search formula and results

Find articles with these terms——"Immunohistochemistry " OR " Tumor mutational burden " OR " gene expression profiling " OR " multiplex immunofluorescence " OR " neoantigen load " OR " Immunofluorescence "

Title, abstract or author-specified keywords：

"Pembrolizumab " OR " Nivolumab " OR " Durvalumab " OR " Toripalimab " OR " Camrelizumab " OR " Atezolizumab " OR " Avelumab " OR " Avelumab " OR " Budigalimab "

Fliter: Research articles

1,659 results till 2.22 Research articles (722)

Eq 2 Cochrane Libarary Search formula and results

#1 MeSH descriptor: [Immune Checkpoint Inhibitors] explode all trees 177

#2 PD L1 Inhibitors or PD L1 Inhibitor or PD-L1 Inhibitors or Programmed Death-Ligand 1 Inhibitors or PD-L1 Inhibitor or Programmed Death Ligand 1 Inhibitors or Pembrolizumab or Nivolumab or Durvalumab or Toripalimab or Camrelizumab or Atezolizumab or Zimberelimab or Avelumab or Budigalimab or pidilizumab or Retifanlimab or Dostarlimab or sugemalimab 8537

#3 #1 or #2 8579

#4 Immunohistochemistry or Tumor mutational burden or RNA sequencing or mutational load or mutational density or mutational landscape or genomic landscape or whole exome sequencing or gene expression profiling or gene signature or EMT stroma-related gene expression or mRNA or circulating tumor DNA or multiplex immunofluorescence or multiplex immunohistochemistry or spatial profiling or neoantigen load or Teff signatures or tumor-infiltrating cells 20131

#5 #3 and #4 1669 1627 trails till 2.22

Eq 3 Pubmed Search formula and results

Query: ((PD-L1 Inhibitors) OR (PD L1 Inhibitors) OR (PD-L1 Inhibitor) OR (PD L1 Inhibitor) OR (Programmed Death-Ligand 1 Inhibitors) OR (Programmed Death Ligand 1 Inhibitors) OR (PD-1-PD-L1 Blockade) OR (Blockade, PD-1-PD-L1) OR (PD 1 PD L1 Blockade) OR (PD-1 Inhibitors) OR (PD 1 Inhibitors) OR (PD-1 Inhibitor) OR (Inhibitor, PD-1) OR (PD 1 Inhibitor) OR (Programmed Cell Death Protein 1 Inhibitor) OR (Programmed Cell Death Protein 1 Inhibitors) OR (Pembrolizumab) OR (Nivolumab) OR (Durvalumab) OR (Toripalimab) OR (Camrelizumab) OR (Atezolizumab) OR (Avelumab) OR (Budigalimab) OR (Zimberelimab) OR (pidilizumab) OR (retifanlimab) OR (dostarlimab) OR (sugemalimab)) AND ((Biomarkers) OR (Marker, Biological) OR (Biological Marker) OR (Biologic Marker) OR (Marker, Biologic) OR (Biological Markers) OR (Biomarker analysis) OR (Immunohistochemistry) OR (Tumor mutational burden) OR (RNA sequencing) OR (mutational load) OR (mutational density) OR (mutational landscape) OR (genomic landscape) OR (whole exome sequencing) OR (gene expression profiling) OR (gene signature) OR (EMT/stroma-related gene expression) OR (mRNA) OR (circulating tumor DNA) OR (multiplex immunofluorescence) OR (multiplex immunohistochemistry) OR (spatial profiling) OR (neoantigen load) OR (Teff signatures) OR (tumor-infiltrating cells)) 19159

Filters: Clinical Study, Clinical Trial, Controlled Clinical Trial, Observational Study, Preprint, Randomized Controlled Trial 1303 till 2.22

Records duplicated remove(n=474)

# Supplementary Figures and Tables

## Supplementary Figures

**Supplementary Figure 1.** Forest Plot by Meta-analysis

I^2^ presents heterogenesity.Abbreviations: Programmed cell death ligand 1 immunohistochemistry (PD-L1 IHC); Tumor mutational burden (TMB); Gene expression profiling (GEP); Microsatellite instability (MSI); Multiplex immunohistochemistry /immunofluorescence (mIHC/IF); Other Immunohistochemistry and hematoxylin-eosin staining (other IHC&HE)

**Supplementary Figure 2.** Deeks’ Funnel Plot by Meta-analysis

P-value presents publication bias. Abbreviations: Programmed cell death ligand 1 immunohistochemistry (PD-L1 IHC); Tumor mutational burden (TMB); Gene expression profiling (GEP); Microsatellite instability (MSI); Multiplex immunohistochemistry /immunofluorescence (mIHC/IF); Other Immunohistochemistry and hematoxylin-eosin staining (other IHC&HE)

**Supplementary Figure 3.** Sub-group Analysis of Combined assays by Meta-analysis

Abbreviations: Programmed cell death ligand 1 immunohistochemistry (PD-L1 IHC); Tumor mutational burden (TMB); Gene expression profiling (GEP); Other Immunohistochemistry and hematoxylin-eosin staining (other IHC&HE)

## Supplementary Tables

| **#** | **Study** | **Author** | **Year** | **Clinical trial** | **Trial phase** | **Antibody** |
| --- | --- | --- | --- | --- | --- | --- |
| 1 | Tumor microenvironment evaluation promotes precise checkpoint immunotherapy of advanced gastric cancer | Dongqiang Zeng | 2021 | NCT02589496 | NA | NA |
| 2 | Tumor Mutational Burden and Efficacy of Nivolumab Monotherapy and in Combination with Ipilimumab in Small-Cell Lung Cancer | Matthew D.Hellmann | 2018 | CHECKMATE-032 | Ⅲ | Nivolumab |
| 3 | TMB and Inflammatory Gene Expression Associated with Clinical Outcomes following Immunotherapy in Advanced Melanoma | F Stephen Hodi | 2021 | CHECKMATE-067 | Ⅲ | Nivolumab |
|  |  |  | 2021 | CHECKMATE-066 | Ⅲ | Nivolumab |
| 4 | Safety, Clinical Activity, and Biological Correlates of Response in Patients with Metastatic Melanoma: Results from a Phase I Trial of Atezolizumab | Omid Hamid | 2019 | NCT01375842 | Ⅰ | Atezolizumab |
| 5 | Safety and clinical activity with an anti-PD-1 antibody JS001 in advanced melanoma or urologic cancer patients | Bixia Tang | 2019 | NCT02836795 | Ⅰ | JS001 |
| 6 | Promising efficacy of SHR-1210, a novel anti-programmed cell death 1 antibody, in patients with advanced gastric and gastroesophageal junction cancer in China | Jing Huang | 2018 | NCI-9742 | Ⅰ | SHR-1210 |
| 7 | Pan-tumor genomic biomarkers for PD-1 checkpoint blockade-based immunotherapy | Razvan Cristescu | 2018 | KEYNOTE-028;  KEYNOTE-012 | IB | Pembrolizumab |
|  |  |  |  | KEYNOTE-012 | IB | Pembrolizumab |
|  |  |  |  | KEYNOTE-001;  KEYNOTE-006 | IB | Pembrolizumab ;Keytruda |
| 8 | Open-label phase II study of the efficacy of nivolumab for cancer of unknown primary Author links open overlay panel | J.Tanizaki | 2022 | UMIN000030649 | Ⅲ | Nivolumab |
| 9 | Impact of zumor mutation burden on nivolumab efficacy in second-line urothelial carcinoma patients: Exploratory analysis of the phase ii checkmate 275 study | M.D. Galsky | 2017 | CheckMate 142 | Ⅱ | Nivolumab |
| 10 | HLA-corrected tumor mutation burden and homologous recombination deficiency for the prediction of response to PD-(L)1 blockade in advanced non-small-cell lung cancer patients | Shim | 2020 | NA | NA | Mutiple Antibodies |
| 11 | Frameshift events predict anti-PD-1/L1 response in head and neck cancer | Glenn J. Hanna | 2018 | NA | NA | NA |
| 12 | First-Line Nivolumab in Stage IV or Recurrent Non-Small-Cell Lung Cancer | Sandra P D'Angelo | 2021 | CHECKMATE-025 | Ⅲ | Nivolumab |
| 13 | Efficacy, Safety, and Biomarkers of Toripalimab in Patients with Recurrent or Metastatic Neuroendocrine Neoplasms: A Multiple-Center Phase Ib Trial | Ming Lu | 2020 | NCT03167853 | IB | Toripalimab |
| 14 | Dose escalation and expansion (phase Ia/Ib) study of GLS-010, a recombinant fully human antiprogrammed death-1 monoclonal antibody for advanced solid tumors or lymphoma | Dan Liu | 2021 | NCT03713905 | IA/IB | GLS-010 |
| 15 | Cobimetinib plus atezolizumab in BRAF wild-type melanoma: primary results from the randomized phase III IMspire170 studyV600 | H Gogas | 2020 | IMspire170 | Ⅲ | Pembrolizumab |
| 16 | Cemiplimab in locally advanced basal cell carcinoma after hedgehog inhibitor therapy: an open-label, multi-centre, single-arm, phase 2 trial | Alexander J Stratigos | 2021 | NCT03132636 | Ⅱ | Cemiplimab |
| 17 | Avelumab in patients with previously treated metastatic Merkel cell carcinoma: long-term data and biomarker analyses from the single-arm phase 2 JAVELIN Merkel 200 trial | Sandra P D'Angelo | 2020 | JAVELIN Merkel 200 | Ⅱ | Avelumab |
| 18 | Association of tumour mutational burden with outcomes in patients with advanced solid tumours treated with pembrolizumab: prospective biomarker analysis of the multicohort, open-label, phase 2 KEYNOTE-158 study | AurélienMarabelleMD | 2020 | KEYNOTE-158 | Ⅱ | Pembrolizumab/PD-1 |
| 19 | A Randomized Phase II Study Comparing Nivolumab with Carboplatin–Pemetrexed for EGFR-Mutated NSCLC with Resistance to EGFR Tyrosine Kinase Inhibitors (WJOG8515L) | Hidetoshi Hayashi | 2022 | jRCTs051180133 | Ⅱ | Nivolumab |
| 20 | Mutational landscape determines sensitivity to PD-1 blockade in non–small cell lung cancer | NAIYER A. RIZVI | 2015 | KEYNOTE-001 | IB | Pembrolizumab |
| 21 | Concurrent High PD-L1 Expression and CD8+ Immune Cell Infiltration Predict PD-1 Blockade Efficacy in Advanced EGFR-Mutant NSCLC Patients | Yukiko Shimoda | 2022 | NA | NA | Multiple antibodies |
| 22 | Efficacy of pembrolizumab in patients with advanced cancer of unknown primary (CUP): a phase 2 non-randomized clinical trial | Kanwal P Raghav | 2022 | NCT02721732 | Ⅱ | Pembrolizumab |
| 23 | Expression of T-Cell Exhaustion Molecules and Human Endogenous Retroviruses as Predictive Biomarkers for Response to Nivolumab in Metastatic Clear Cell Renal Cell Carcinoma | Miriam Ficial | 2021 | CHECKMATE-025 | Ⅲ | Nivolumab |
| 24 | irRECIST for the Evaluation of Candidate Biomarkers of Response to Nivolumab in Metastatic Clear Cell Renal Cell Carcinoma: Analysis of a Phase II Prospective Clinical Trial | Jean-Christophe Pignon | 2019 | CHECKMATE-010 | Ⅱ | Nivolumab |
| 25 | Serum Antibody Against NY-ESO-1 and XAGE1 Antigens Potentially Predicts Clinical Responses to Anti–Programmed Cell Death-1 Therapy in NSCLC | YoshihiroOhue | 2019 | NA | NA | Pembrolizumab |
| 26 | ZEBRA: A Multicenter Phase II Study of Pembrolizumab in Patients with Advanced Small-Bowel Adenocarcinoma | Katrina S Pedersen | 2021 | NCT02949219 | Ⅱ | Pembrolizumab |
| 27 | Squamous differentiation is a potential biomarker predicting tumor progression in patients treated with pembrolizumab for urothelial carcinoma | Yu Miyama | 2021 | NA | NA | Pembrolizumab/  PD-1 |
| 28 | Retrospective Evaluation of the Use of Pembrolizumab in Malignant Mesothelioma in a Real-World Australian Population | Tamkin Ahmadzada BE | 2020 | NA | NA | Pembrolizumab |
| 29 | Phase 2 study of pembrolizumab in patients with advanced rare cancers | Aung Naing | 2020 | NCT02721732 | Ⅱ | Pembrolizumab |
| 30 | Long-term efficacy and predictive correlates of response to nivolumab in Japanese patients with esophageal cancer | Ken Kato | 2020 | ONO-4538-07 | Ⅱ |  |
| 31 | Long-term Clinical Outcomes and Biomarker Analyses of Atezolizumab Therapy for Patients With Metastatic Triple-Negative Breast Cancer: A Phase 1 Study | Leisha A. Emens | 2019 | NCT01375842 | Ⅰ | Atezolizumab |
| 32 | Association of Baseline and Pharmacodynamic Biomarkers With Outcomes in Patients Treated With the PD-1 Inhibitor Budigalimab | Stacie L Lambert | 2022 | NCT03000257 | Ⅰ | Budigalimab |
| 33 | Safety and Efficacy of Durvalumab and Tremelimumab Alone or in Combination in Patients with Advanced Gastric and Gastroesophageal Junction Adenocarcinoma | Ronan J. Kelly | 2019 | NCT02340975 | Ⅱ | Durvalumab or Tremelimumab |
| 34 | Safety and Efficacy of Pembrolizumab Monotherapy in Patients With Previously Treated Advanced Gastric and Gastroesophageal Junction Cancer: Phase 2 Clinical KEYNOTE-059 Trial | Charles S. Fuchs | 2018 | KEYNOTE-059 | Ⅱ | Pembrolizumab |
| 35 | Phase II Study of Avelumab in Patients With Mismatch Repair Deficient and Mismatch Repair Proficient Recurrent/Persistent Endometrial Cancer | Panagiotis A Konstantinopoulos | 2019 | NCT02912572 | Ⅱ | Avelumab |
| 36 | PD-1 Blockade in Advanced Adrenocortical Carcinoma | Nitya Raj | 2020 | NCT02673333 | Ⅱ | Pembrolizumab |
| 37 | Nivolumab in patients with metastatic DNA mismatch repair-deficient or microsatellite instability-high colorectal cancer (CheckMate 142): an open-label, multicentre, phase 2 study | Dr Michael J Overman | 2017 | CheckMate 142 | Ⅱ | Nivolumab |
| 38 | Comprehensive molecular characterization of clinical responses to PD-1 inhibition in metastatic gastric cancer | Seung Tae Kim | 2018 | NCT02589496 | Ⅱ | Pembrolizumab |
| 39 | Atezolizumab with or without cobimetinib versus regorafenib in previously treated metastatic colorectal cancer (IMblaze370): a multicentre, open-label, phase 3, randomised, controlled trial | CathyEngMD | 2019 | IMblaze370 | Ⅲ | Atezolizumab |
| 40 | Association of serine/threonine kinase 11 mutations and response to programmed cell death 1 inhibitors in metastatic gastric cancer | MinsukKwon | 2020 | NA | NA | Pembrolizumab  or Nivolumab |
| 41 | Interferon Gamma Messenger RNA Signature in Tumor Biopsies Predicts Outcomes in Patients with Non–Small Cell Lung Carcinoma or Urothelial Cancer Treated with Durvalumab | Brandon W Higgs | 2018 | CD-ON-MEDI4736-1108 | IB/II | Durvalumab |
| 42 | Clinical activity and molecular correlates of response to atezolizumab alone or in combination with bevacizumab versus sunitinib in renal cell carcinoma | David F. McDermott, | 2019 | IMmotion150 | Ⅱ | Atezolizumab |
| 43 | Nivolumab in metastatic urothelial carcinoma after platinum therapy (CheckMate 275): a multicentre, single-arm, phase 2 trial | ProfPadmaneeSharmaMD | 2017 | NCT02387996 | Ⅱ | Nivolumab |
| 44 | Intratumoral CD39CD8 T Cells Predict Response to Programmed Cell Death Protein-1 or Programmed Death Ligand-1 Blockade in Patients With NSCLC | Joe Yeong M.B.B.S | 2021 | NA | NA | Multiple antibodies |
| 45 | Safety, efficacy and tumor mutational burden as a biomarker of overall survival benefit in chemo-refractory gastric cancer treated with toripalimab, a PD-1 antibody in phase Ib/II clinical trial NCT02915432 | F.Wang | 2019 | NCT02915432 | Ib/II | Toripalimab |
| 46 | Pan-cancer analysis of longitudinal metastatic tumors reveals genomic alterations and immune landscape dynamics associated with pembrolizumab sensitivity | S Y Cindy Yang | 2021 | NCT02644369 | Ⅱ | Pembrolizumab |
| 47 | Atezolizumab Treatment of Tumors with High Tumor Mutational Burden from MyPathway, a Multicenter, Open-Label, Phase IIa Multiple Basket Study | Claire F. Friedman | 2022 | NCT02091141 | ⅡA | Atezolizumab |
| 48 | EMT- and stroma-related gene expression and resistance to PD-1 blockade in urothelial cancer | wangli | 2018 | CHECKMATE-275 | Ⅱ | Nivolumab |
| 49 | HLA-I diversity and tumor mutational burden by comprehensive next-generation sequencing as predictive biomarkers for the treatment of non-small cell lung cancer with PD-(L)1 inhibitors | Kristof Cuppens | 2022 | NA | NA | Multiple antibodies |

**Supplementary Table 1.** Included article

| **#** | **Assay** | **Method** | **Threshold or Positve Range** | **Tumor Type** | **Study size** | **TP** | **FN** | **FP** | **TN** |
| --- | --- | --- | --- | --- | --- | --- | --- | --- | --- |
| 1* | TMB | non-synonymous SNVs | ≥400 | Gastric cancer | 45 | 2 | 10 | 22 | 11 |
| 2 | PD-L1 IHC | DAKO 28-8 | TPS 1% | Small-cell lung cancer | 75 | 1 | 9 | 10 | 55 |
| 2 | TMB | somatic missense mutations SNPs | tertiles ≥high | Small-cell lung cancer | 133 | 10 | 5 | 27 | 91 |
| 3 | GEP | 4-gene inflammatory CD274, CD8A, LAG3, and STAT1 | >median | Melanoma | 97 | 30 | 19 | 16 | 32 |
| 3 | combined assays | GEP+TMB | ** | Melanoma | 75 | 18 | 19 | 6 | 32 |
| 3 | TMB | missense mutations | >median | Melanoma | 176 | 52 | 30 | 33 | 61 |
| 3 | other IHC&HE | C8/144B | CD8, median | Melanoma | 67 | 20 | 9 | 14 | 24 |
| 3 | combined assays | TMB+CD8 | ** | Melanoma | 67 | 13 | 16 | 5 | 33 |
| 3 | PD-L1 IHC | Dako 28-8 | TC≥5% | Melanoma | 176 | 54 | 28 | 33 | 61 |
| 3 | PD-L1 IHC | DAKO 28-8 | TC≥5% | Melanoma | 52 | 14 | 8 | 11 | 19 |
| 3 | TMB | missense mutations | >median | Melanoma | 52 | 14 | 8 | 9 | 21 |
| 4 | PD-L1 IHC | SP142 | IC≥1 | Melanoma | 37 | 9 | 3 | 13 | 12 |
| 4 | TMB | targeted genomic profiling by Foundation Medicine | 16 mutations/Mb | Melanoma | 22 | 6 | 0 | 5 | 11 |
|  | other IHC&HE | CD8 IHC (SP16 clone) | post medium | Melanoma | 38 | 8 | 4 | 12 | 14 |
| 5 | PD-L1 IHC | SP142 | TC≥5% | Multiple cancers | 28 | 7 | 0 | 9 | 12 |
| 5 | TMB | somatic mutations including coding base substitution and INDELs per mega-base of the panel sequences examined | 6,median | Multiple cancers | 23 | 5 | 2 | 6 | 10 |
| 5 | other IHC&HE | CD8 Clone 4B11, BioRad, Cat# MCA 1817 T | No description | Multiple cancers | 28 | 7 | 0 | 15 | 6 |
| 5 | combined assays | PDL1+TMB | ** | Multiple cancers | 21 | 5 | 1 | 4 | 11 |
| 6 | PD-L1 IHC | 6E8 antibody | TC≥1% | Gastroesophageal junction and gastric cancer | 30 | 1 | 6 | 4 | 19 |
| 6 | TMB | GATK Best Practices Pipeline post medium | ≥meidan(80/cell) | Gastroesophageal junction and gastric cancer | 20 | 4 | 2 | 6 | 8 |
| 7 | GEP | CCL5, CD27, CD274 (PD-L1), CD276 (B7-H3), CD8A, CMKLR1, CXCL9, CXCR6, HLA-DQA1, HLA-DRB1, HLA-E, IDO1, LAG3, NKG7, PDCD1LG2 (PDL2), PSMB10, STAT1 and TIGIT | ≥66.6% | Multiple cancers | 113 | 15 | 1 | 53 | 44 |
| 7 | TMB | somatic nonsynonymous SNVs | > 100 mutations per exome | Multiple cancers | 113 | 11 | 5 | 25 | 72 |
| 7 | combined assays | TMB+GEP | ** | Multiple cancers | 113 | 10 | 6 | 17 | 80 |
| 7 | PD-L1 IHC | Dako PD-L1 IHC 22C3 pharmDx | CPS≥1% | Multiple cancers | 74 | 9 | 0 | 34 | 31 |
| 7 | combined assays | TMB+PDL1 | ** | Multiple cancers | 74 | 7 | 2 | 13 | 52 |
| 7 | GEP | CCL5, CD27, CD274 (PD-L1), CD276 (B7-H3), CD8A, CMKLR1, CXCL9, CXCR6, HLA-DQA1, HLA-DRB1, HLA-E, IDO1, LAG3, NKG7, PDCD1LG2 (PDL2), PSMB10, STAT1 and TIGIT | ≥66.6% | HNSCC | 105 | 20 | 1 | 55 | 29 |
| 7 | TMB | somatic nonsynonymous SNVs | > 100 mutations per exome | HNSCC | 107 | 15 | 6 | 39 | 47 |
| 7 | combined assays | TMB+GEP | ** | HNSCC | 107 | 8 | 13 | 18 | 68 |
| 7 | PD-L1 IHC | Dako PD-L1 IHC 22C3 pharmDx | CPS≥1% | HNSCC | 107 | 19 | 2 | 70 | 16 |
| 7 | combined assays | TMB+PDL1 | ** | HNSCC | 107 | 14 | 7 | 33 | 53 |
| 7 | GEP | CCL5, CD27, CD274 (PD-L1), CD276 (B7-H3), CD8A, CMKLR1, CXCL9, CXCR6, HLA-DQA1, HLA-DRB1, HLA-E, IDO1, LAG3, NKG7, PDCD1LG2 (PDL2), PSMB10, STAT1 and TIGIT | ≥66.6% | Melanoma | 86 | 32 | 6 | 31 | 17 |
| 7 | TMB | somatic nonsynonymous SNVs | > 100 mutations per exome | Melanoma | 89 | 31 | 7 | 24 | 27 |
| 7 | combined assays | TMB+GEP | ** | Melanoma | 89 | 29 | 9 | 25 | 26 |
| 7 | PD-L1 IHC | Dako PD-L1 IHC 22C3 pharmDx | CPS≥1% | Melanoma | 89 | 35 | 3 | 43 | 8 |
| 7 | combined assays | TMB+PDL1 | ** | Melanoma | 89 | 28 | 10 | 21 | 30 |
| 8 | PD-L1 IHC | DAKO 28-8 | TC 1% | Cancer of unknown primary | 51 | 10 | 2 | 21 | 18 |
| 8 | TMB | NGS panel | ≥7.75 mut/Mb | Cancer of unknown primary | 46 | 7 | 3 | 16 | 20 |
| 8 | other IHC&HE | DAKO 28-8 | TIL≥median | Cancer of unknown primary | 50 | 10 | 2 | 23 | 15 |
| 8 | MSI | MSI-high on the basis of the presence of two or more unstable markers | MSS/MSIH | Cancer of unknown primary | 42 | 2 | 8 | 0 | 32 |
| 9* | TMB | missense somatic mutations per tumor | tertiles high≥167 | Cancer of unknown primary | 139 | 23 | 5 | 70 | 41 |
| 10 | PD-L1 IHC | Dako PD-L1 IHC 22C3 pharmDx | ≥50% | NSCLC | 198 | 24 | 37 | 23 | 114 |
| 10 | TMB | nonsynonymous alteration (single-nucleotide variations or indels | 143 mutations/Mb | NSCLC | 198 | 31 | 30 | 45 | 92 |
| 10 | combined assays | HLA-LOH+TMB | ** | NSCLC | 198 | 22 | 39 | 15 | 122 |
| 10 | GEP | HLA analysis silico neoantigen prediction MuPeXI | HLA-INTACT/LOH | NSCLC | 198 | 17 | 44 | 37 | 100 |
| 11 | TMB | nonsynonymous somatic mutations that occur per megabase of exonic sequence data | 10mut/Mb | SCCHN | 71 | 6 | 6 | 8 | 51 |
| 11 | PD-L1 IHC | PD-L1 rabbit mAb 13684 | TC scored positive if 1 or higher or 50% or higher | SCCHN | 42 | 9 | 8 | 6 | 19 |
| 12 | PD-L1 IHC | PD-L1 73-10 | TC 50% | NSCLC | 158 | 26 | 21 | 31 | 80 |
| 12 | TMB | NSSV | 2 NSSV/Mb | NSCLC | 158 | 22 | 25 | 25 | 86 |
| 13 | PD-L1 IHC | SP142 | TC 1% | Neuroendocrine neoplasms | 36 | 6 | 2 | 6 | 22 |
| 13 | TMB | somatic mutations including coding base substitution and INDELs | 90% (9.9 mutations/Mb) | Neuroendocrine neoplasms | 35 | 3 | 5 | 1 | 26 |
| 13 | MSI | MANTIS with default parameter | MSI-H | Neuroendocrine neoplasms | 35 | 1 | 7 | 1 | 26 |
| 14 | PD-L1 IHC | SP263 | Multiple threshold | Solid tumors or lymphoma | 193 | 25 | 16 | 54 | 98 |
| 14 | TMB | somatic mutations per mega-base (Mb) | tTMB 4.3 mutations/Mb | Solid tumors or lymphoma | 97 | 15 | 3 | 33 | 46 |
| 15 | PD-L1 IHC | SP142 | IC 1% | BRAF wild-type melanoma | 205 | 49 | 15 | 88 | 53 |
| 15 | TMB | FoundationOne | ≥10 mutations/Mb | BRAF wild-type melanoma | 164 | 37 | 17 | 53 | 57 |
| 16 | PD-L1 IHC | 22C3 | TC 1% | Basal cell carcinoma | 50 | 4 | 9 | 11 | 26 |
| 16 | TMB | somatic SNVs and indels in the coding regions of targeted genes per megabase of analysed genomic sequence | ≥10 mutations/Mb | Basal cell carcinoma | 56 | 16 | 2 | 28 | 10 |
| 17 | PD-L1 IHC | PD-L1 73-10 | TC 1% | Merkel cell carcinoma | 73 | 21 | 3 | 36 | 13 |
| 17 | combined assays | TMB+PDL1 | ** | Merkel cell carcinoma | 36 | 5 | 7 | 4 | 20 |
| 17 | TMB | non-synonymous somatic variants per megabase (NSSV/Mb) | ≥2 NSSV/Mb | Merkel cell carcinoma | 36 | 5 | 7 | 6 | 18 |
| 17 | other IHC&HE | clone C8/144B | CD8,median | Merkel cell carcinoma | 30 | 9 | 1 | 11 | 9 |
| 17 | combined assays | TMB+CD8+ | ** | Merkel cell carcinoma | 30 | 5 | 5 | 1 | 19 |
| 18 | PD-L1 IHC | 22C3 | CPS≥1 | Solid tumours | 754 | 58 | 14 | 393 | 289 |
| 18 | TMB | FoundationOne CDx | ≥10 Mut/Mb | Solid tumours | 790 | 30 | 43 | 72 | 645 |
| 18 | combined assays | TMB+PDL1 IHC |  | Solid tumours | 754 | 24 | 48 | 44 | 638 |
| 19 | PD-L1 IHC | DAKO 28-8 | TPS 1% | EGFR-mutated NSCLC | 23 | 1 | 6 | 2 | 14 |
| 19 | TMB | Ion Reporter version 5.10 | ≥post medium | EGFR-mutated NSCLC | 25 | 2 | 2 | 9 | 12 |
| 20 | PD-L1 IHC | 22C3 | TC 50% | NSCLC | 30 | 11 | 1 | 13 | 5 |
| 20 | TMB | nonsynonymous mutation burden | ≥178 Mut/Mb | NSCLC | 34 | 8 | 4 | 9 | 13 |
| 20 | other IHC&HE | neoantigen prediction | medium | NSCLC | 34 | 11 | 3 | 5 | 15 |
| 21 | PD-L1 IHC | clone 22C3 | TPS 50% | EGFR-mutant NSCLC | 39 | 5 | 2 | 9 | 23 |
| 21 | other IHC&HE | clone 4B | CD8 IC >205 | EGFR-mutant NSCLC | 37 | 6 | 1 | 6 | 24 |
| 21 | combined assays | PDL1+CD8 | ** | EGFR-mutant NSCLC | 35 | 5 | 2 | 1 | 27 |
| 22 | PD-L1 IHC | Merck 22C3 | IC≥2 | Cancer of unknown primary | 25 | 4 | 1 | 5 | 15 |
| 22 | other IHC&HE | HE | TIL 3 | Cancer of unknown primary | 25 | 4 | 1 | 5 | 15 |
| 22 | combined assays | PDL1+TIL | ** | Cancer of unknown primary | 25 | 5 | 0 | 8 | 12 |
| 23 | PD-L1 IHC | Dako | TC 1% | Clear cell renal cell carcinoma | 111 | 6 | 21 | 6 | 78 |
| 23 | mIHC/IF | PerkinElmer/Akoya Biosciences Cat# NEL871001KT and Cat# DS9800 | CD8+ PD-1+TIM-3-LAG-3- TIC | Clear cell renal cell carcinoma | 116 | 11 | 18 | 13 | 74 |
| 23 | combined assays | PDL1+TIL | ** | Clear cell renal cell carcinoma | 111 | 6 | 21 | 6 | 78 |
| 24 | PD-L1 IHC | Dako (clone 28–8) | TC 1% | Clear cell renal cell carcinoma | 138 | 9 | 23 | 10 | 96 |
| 24 | other IHC&HE | clone D7U8C | PD-L2 TC 1% | Clear cell renal cell carcinoma | 127 | 11 | 19 | 35 | 62 |
| 24 | mIHC/IF | CD8, PD-1, TIM-3 and LAG-3 multiplex immunofluorescence Opal tyramide signal system from Perkin Elmer | CD8+ PD-1+TIM-3-LAG-3- TIC, 36% | Clear cell renal cell carcinoma | 98 | 21 | 0 | 53 | 24 |
| 24 | combined assays | PDL1+MIHC | ** | Clear cell renal cell carcinoma | 97 | 6 | 15 | 5 | 71 |
| 25 | PD-L1 IHC |  | TPS≥5% | NSCLC | 70 | 18 | 6 | 18 | 28 |
| 25 | combined assays | PDL1+NY-ESO-1 / XAGE1 T cell | ** | NSCLC | 70 | 9 | 15 | 3 | 43 |
| 25 | other IHC&HE | NY-ESO-1 and XAGE1 Abs | CT antibody post medium | NSCLC | 72 | 14 | 10 | 4 | 44 |
| 25 | combined assays | CD8+CT antibody | ** | NSCLC | 72 | 8 | 17 | 1 | 46 |
| 25 | other IHC&HE | CD8 | CD8 + T-cell infiltration post medium | NSCLC | 72 | 12 | 13 | 13 | 34 |
| 26 | PD-L1 IHC | Merck 22C3 | No description | Small-bowel adenocarcinoma | 25 | 3 | 1 | 8 | 13 |
| 26 | MSI | NGS(Tempus xT) or IHC | MSI-H/other | Small-bowel adenocarcinoma | 33 | 2 | 2 | 1 | 28 |
| 26 | other IHC&HE | CD8+ TIL 2+ | CD8+ TIL | Small-bowel adenocarcinoma | 25 | 4 | 0 | 9 | 12 |
| 27 | PD-L1 IHC | clone SP263 | IC 5% | Urothelial carcinoma | 33 | 4 | 6 | 8 | 15 |
| 27 | other IHC&HE | CD204+ macrophages cloneSRA-E5 | ≥median | Urothelial carcinoma | 33 | 6 | 4 | 12 | 11 |
| 28 | PD-L1 IHC | E1L3N clone | TPS≥1 | Mesothelioma | 76 | 7 | 5 | 24 | 40 |
| 28 | other IHC&HE | CD3 LN10 clone | TC 5% | Mesothelioma | 76 | 10 | 2 | 26 | 38 |
| 29 | PD-L1 IHC |  | H-score >42.5 | Multiple cancers | 79 | 6 | 13 | 9 | 51 |
| 29 | other IHC&HE | HE | TILS 3 | Multiple cancers | 79 | 5 | 14 | 14 | 46 |
| 30 | PD-L1 IHC | 28‐8 | TC 5% | Esophageal cancers | 34 | 5 | 2 | 7 | 20 |
| 30 | other IHC&HE | Monoclonal mouse anti‐human Ab | TILS >63.75% | Esophageal cancers | 34 | 4 | 3 | 14 | 13 |
| 30 | mIHC/IF | Monoclonal mouse anti‐human Ab | CD8 >50%,TILS >63.5% | Esophageal cancers | 31 | 2 | 5 | 8 | 16 |
| 31 | PD-L1 IHC | SP142 | IC≥1 | MTNBC | 96 | 9 | 2 | 55 | 30 |
| 31 | other IHC&HE | CD8 IHC (C8/144B) | ≥median | MTNBC | 90 | 7 | 3 | 40 | 40 |
| 32 | PD-L1 IHC | Dako 28-8 | TC 1% | NSCLC | 33 | 2 | 4 | 3 | 24 |
| 32 | other IHC&HE | Dako C8 / 144B | CD8 T-cell infiltration≥15% | NSCLC | 33 | 2 | 5 | 3 | 23 |
| 33 | PD-L1 IHC | SP263 | CPS 1% | Gastroesophageal junction and gastric cancer | 20 | 0 | 1 | 9 | 10 |
| 33 | MSI | MSIsensor | MSI-H | Gastroesophageal junction and gastric cancer | 22 | 0 | 1 | 1 | 20 |
| 34 | PD-L1 IHC | 22C3 | CPS 0 | Gastroesophageal junction and gastric cancer | 216 | 23 | 7 | 105 | 81 |
| 34 | MSI |  | MSI-H | Gastroesophageal junction and gastric cancer | 145 | 4 | 15 | 1 | 125 |
| 35 | PD-L1 IHC |  | TC 1% | Recurrent/persistent endometrial cancer | 25 | 0 | 5 | 7 | 13 |
| 35 | MSI | Oncopanel;Dana Farber | MMRD/MMRPnonPOLE(IHC+TMB+GEM),subtype | Recurrent/persistent endometrial cancer | 26 | 4 | 1 | 8 | 13 |
| 36 | PD-L1 IHC | QualTek Molecular Laboratories | TC 1% | Adrenocortical carcinomas | 34 | 2 | 7 | 5 | 20 |
| 36 | MSI | MSIsensor | MSI sensor score ≥ 10 or an MSI sensor score ≥ 3 with tumor mutation burden (TMB) > 10 mutations/megabase (mut/Mb) | Adrenocortical carcinomas | 38 | 2 | 6 | 4 | 26 |
| 37 | MSI | dMMR/MSI-H IHC/PCR | zero, one, or two or more markers were identified as microsatellite-stable, MSI-low, and MSI-H | Colorectal cancer | 67 | 19 | 3 | 34 | 11 |
| 37 | PD-L1 IHC | Dako 28-8 | TC 1 | Colorectal cancer | 68 | 6 | 13 | 15 | 34 |
| 38 | PD-L1 IHC | 22C3 | CPS≥1 | Metastatic gastric cancer | 55 | 14 | 0 | 14 | 27 |
| 38 | GEP | Exome regions defined by ACRG | EMT subtype | Metastatic gastric cancer | 39 | 0 | 10 | 6 | 23 |
| 38 | MSI | IHC and BAT-25, BAT-26, NR-21, NR-24, and NR-27 | MSI-H | Metastatic gastric cancer | 49 | 6 | 8 | 0 | 35 |
| 39 | PD-L1 IHC | SP142 | IC 1% | Colorectal cancer | 77 | 1 | 1 | 34 | 41 |
| 39 | MSI | Foundation Medicines | ** | Colorectal cancer | 77 | 1 | 1 | 2 | 73 |
| 40 | PD-L1 IHC | 22C3 | CPS≥1 | Gastric cancer | 46 | 8 | 8 | 3 | 27 |
| 40 | MSI | BAT-25, BAT-26, NR-21, NR-24, and NR-27) | ≥2 MSIH | Gastric cancer | 56 | 7 | 14 | 2 | 33 |
| 41 | GEP | 4-gene interferon-γ signature | ≥75% | NSCLC | 97 | 10 | 2 | 38 | 47 |
| 41 | PD-L1 IHC | SP263 | TC 25% | NSCLC | 285 | 41 | 7 | 124 | 113 |
| 41 | GEP | 4-gene interferon-γ signature | ≥75% | Urothelial cancer | 62 | 10 | 7 | 5 | 40 |
| 41 | PD-L1 IHC | SP263 | TC 25% | Urothelial cancer | 100 | 23 | 2 | 38 | 37 |
| 42 | PD-L1 IHC | SP142 | 5% | Renal cell carcinoma | 103 | 15 | 11 | 39 | 38 |
| 42 | GEP | Teff TruSeq RNA Access technology | post medium | Renal cell carcinoma | 86 | 14 | 6 | 32 | 34 |
| 43 | PD-L1 IHC | DAKO 28-8 | TC 1% | Urothelial carcinoma | 216 | 29 | 23 | 72 | 92 |
| 43 | GEP | 25-gene interferon-γ signature | tertiles≥high | Urothelial carcinoma | 177 | 20 | 19 | 39 | 99 |
| 44 | PD-L1 IHC | SP263 | TPS 1% | NSCLC | 29 | 3 | 0 | 11 | 15 |
| 44 | mIHC/IF | Opal Multiplex fIHC kit | CD39CD8 31.5 | NSCLC | 29 | 3 | 0 | 3 | 23 |
| 45 | TMB | somatic mutations per mega-base (Mb) | 12 Mut/Mb | Gastric cancer | 54 | 4 | 3 | 8 | 39 |
| 45 | MSI |  | MSI-H | Gastric cancer | 55 | 1 | 6 | 0 | 48 |
| 46 | TMB | non-synonymous mutations per million bases | 10 Mut/Mb | Multiple cancers | 45 | 5 | 12 | 1 | 27 |
| 46 | GEP | percent genome with copy number alterations | ≥66.6% | Multiple cancers | 45 | 2 | 15 | 13 | 15 |
| 47 | TMB | FoundationOne or FoundationOne CDx [F1(CDx)] | 16mut/Mb | Multiple cancers | 90 | 16 | 1 | 26 | 47 |
| 47 | MSI | FoundationOne CDx [F1(CDx)] | MSI-H | Multiple cancers | 85 | 6 | 9 | 5 | 65 |
| 47 | muti-assay | TMB+MSIH | ** | Multiple cancers | 85 | 6 | 9 | 5 | 65 |
| 48 | other IHC&HE | C8 / 144B | CD8 infiltration≥median | Urothelial cancer | 214 | 27 | 13 | 80 | 94 |
| 48 | GEP | EMT / Stroma_core | ≥median | Urothelial cancer | 214 | 17 | 22 | 90 | 85 |
| 48 | combined assays | GEP+CD8 | ** | Urothelial cancer | 214 | 11 | 28 | 54 | 121 |
| 49 | TMB | nonsynonymous mutations | 10 mutations/Mb | NSCLC | 126 | 27 | 18 | 28 | 53 |
| 49 | GEP | HLA-1 diversity | upper 15th percentile (P15) | NSCLC | 126 | 20 | 25 | 25 | 56 |
| 49 | combined assays | TMB+GEP | ** | NSCLC | 126 | 12 | 33 | 7 | 74 |

**Supplementary Table 2.** Details of Included Studies

*Studies based on same clinical trials were combined in our analysis

** The Threshold or positve range for combined assays were positive when the two testing tests were positive.

Abbreviations: True positives (TP); False negatives (FN); False positives (FP); True negatives (TN); Mutations (Mut); Proficient mismatch repair (pMMR) proteins; Deficient mismatch repair (dMMR); Next-generation sequencing (NGS); Tumor cell proportion score (TPS); Tumor cell (TC); Combined positive score (CPS); Immune cell (IC); Programmed cell death ligand 1 immunohistochemistry (PD-L1 IHC); Tumor mutational burden (TMB); Gene expression profiling (GEP); Microsatellite instability (MSI); Multiplex immunohistochemistry /immunofluorescence (mIHC/IF); Other Immunohistochemistry and hematoxylin-eosin staining (other IHC&HE); Non-small cell lung cancer (NSCLC); Squamous cell carcinoma of the head and neck (SCCHN)

**Supplementary Table 3. QUADAS-C**
