# Supplementary material for: Comparison of different predictive biomarker testing assays for PD-1/PD-L1 checkpoint inhibitors response: a systematic review and network meta-analysis
Source: Front Immunol. 2023 Sep 26;14:1265202. doi: 10.3389/fimmu.2023.1265202 (PMC10562577; doi:10.3389/fimmu.2023.1265202)
Supplement: Supplementary file 2 [file Image_1.pdf]

Supplementary Figure 1. Forest Plot by Meta-analysis

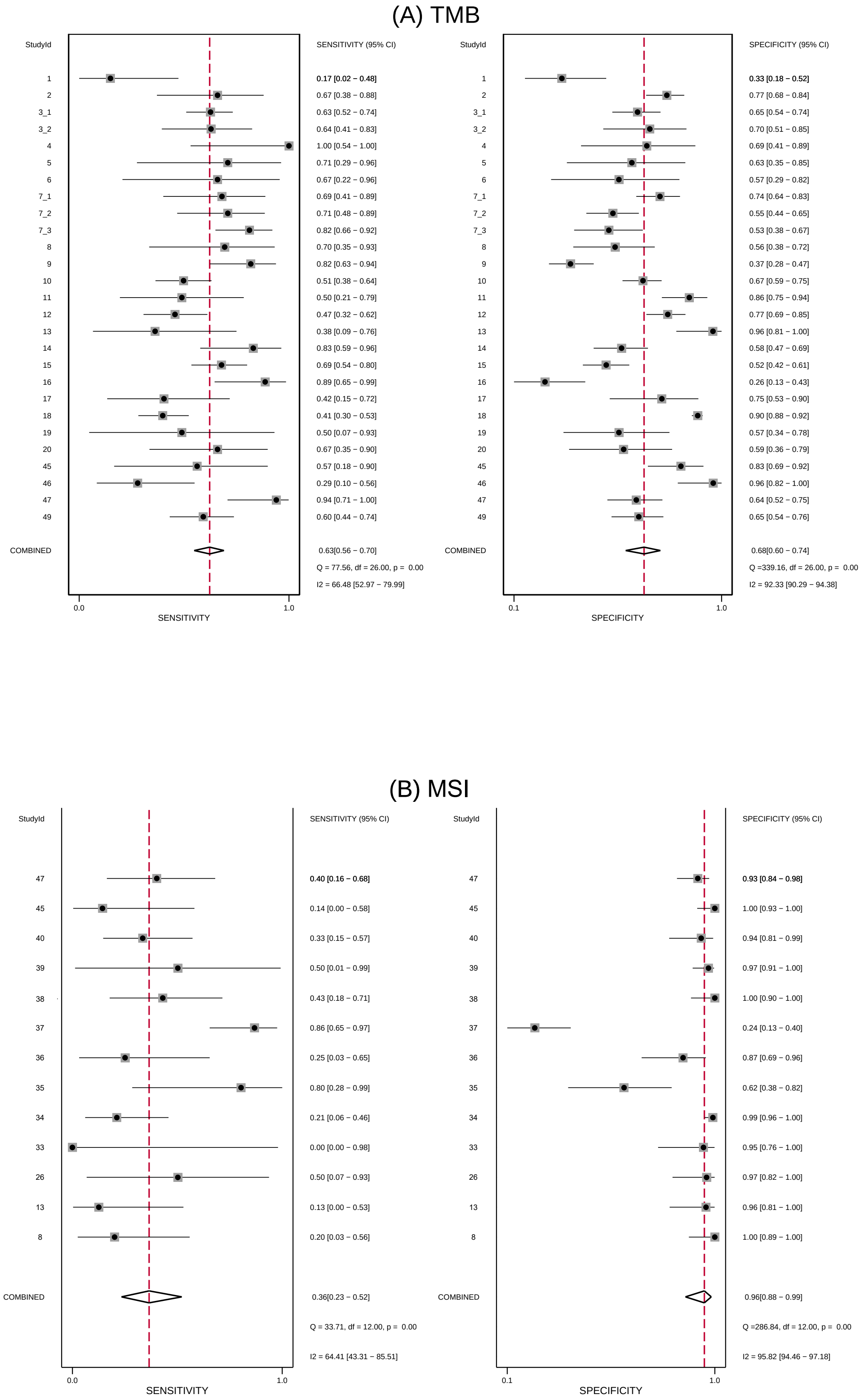

(C) mIHC

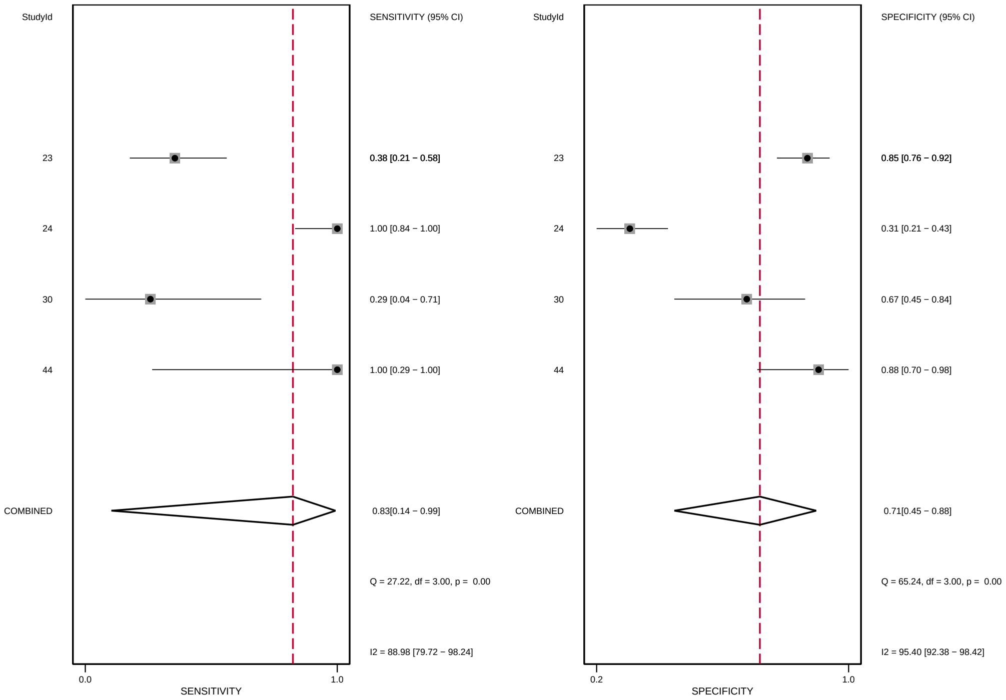

(D) other IHC

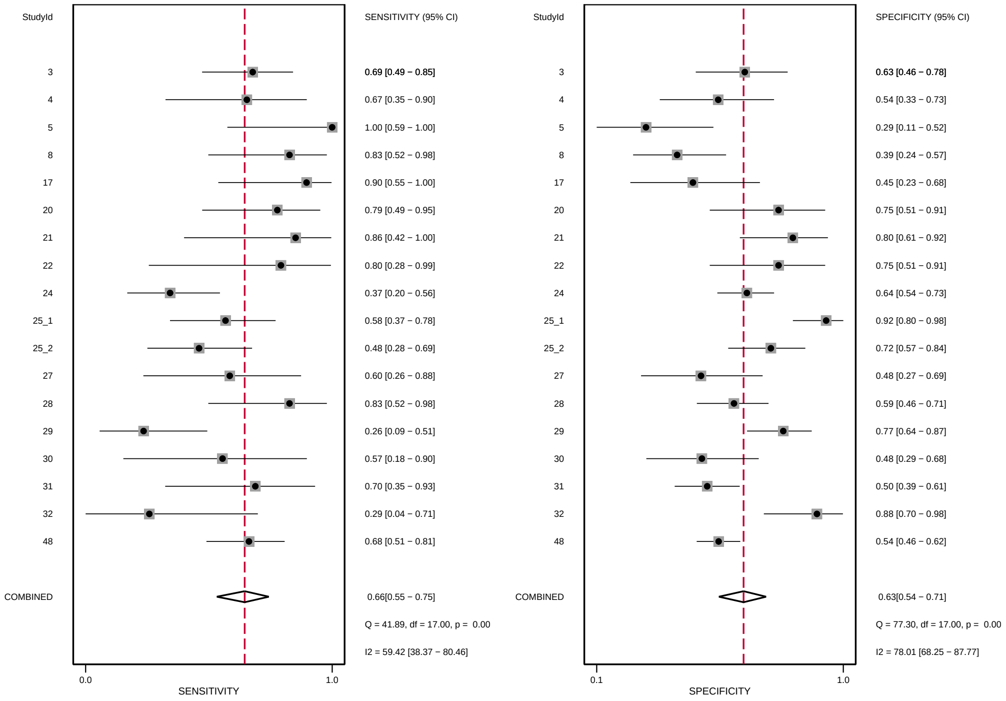

(E) GEP

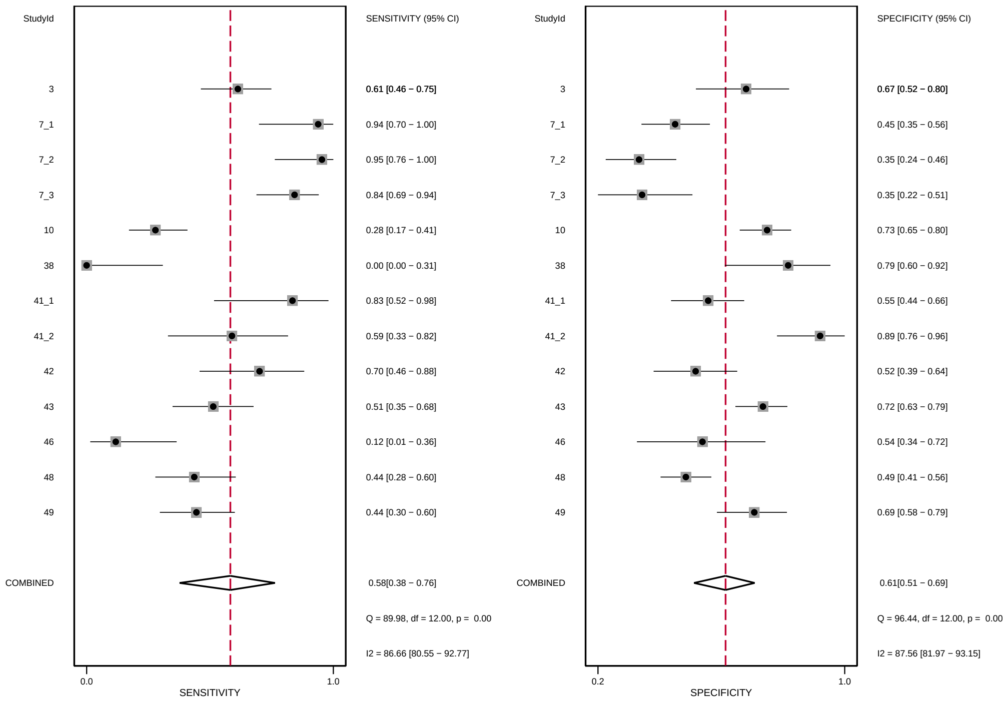

(F) combined assays

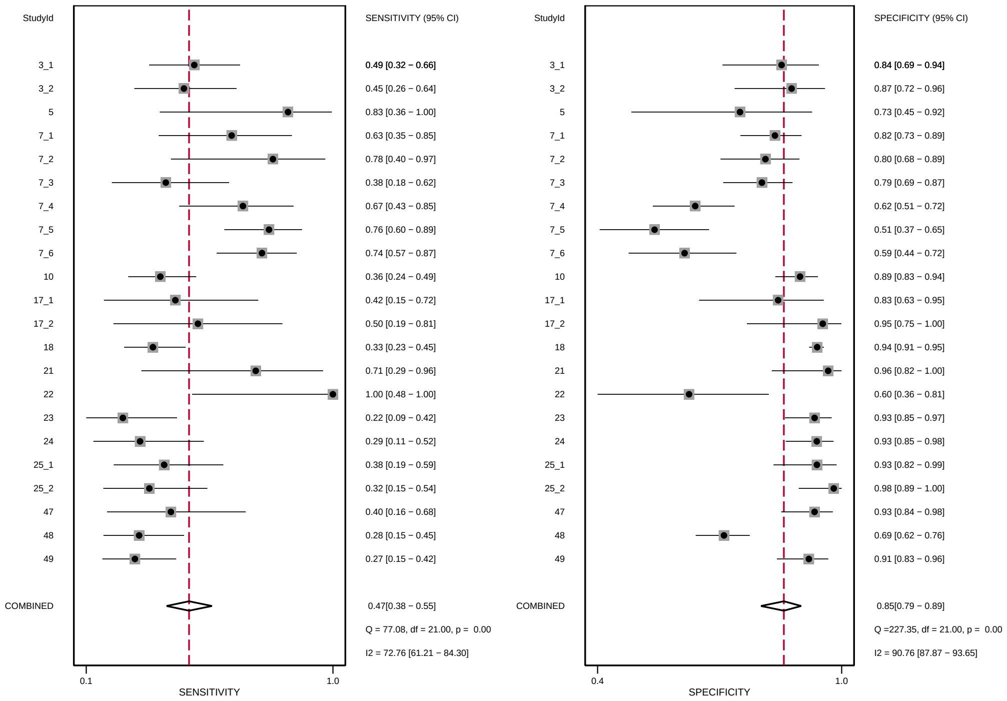

(G) PD-L1 IHC

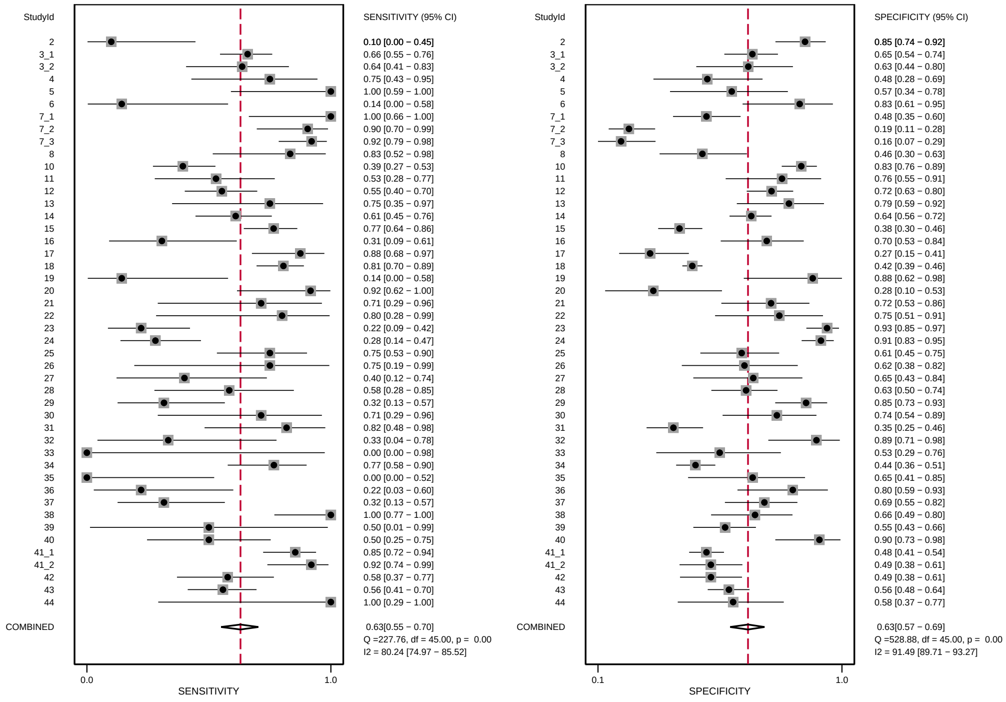

I^2 presents heterogenesity.

Abbreviations: Programmed cell death ligand 1 immunohistochemistry (PD-L1 IHC); Tumor mutational burden (TMB); Gene expression profiling (GEP); Microsatellite instability (MSI); Multiplex immunohistochemistry /immunofluorescence (mIHC/IF); Other Immunohistochemistry and hematoxylin-eosin staining (other IHC&HE)
