# Supplementary material for: Comparison of different predictive biomarker testing assays for PD-1/PD-L1 checkpoint inhibitors response: a systematic review and network meta-analysis
Source: Front Immunol. 2023 Sep 26;14:1265202. doi: 10.3389/fimmu.2023.1265202 (PMC10562577; doi:10.3389/fimmu.2023.1265202)
Supplement: Supplementary file 3 [file Image_2.pdf]

Supplementary Figure 2. Deeks' Funnel Plot by Meta-analysis

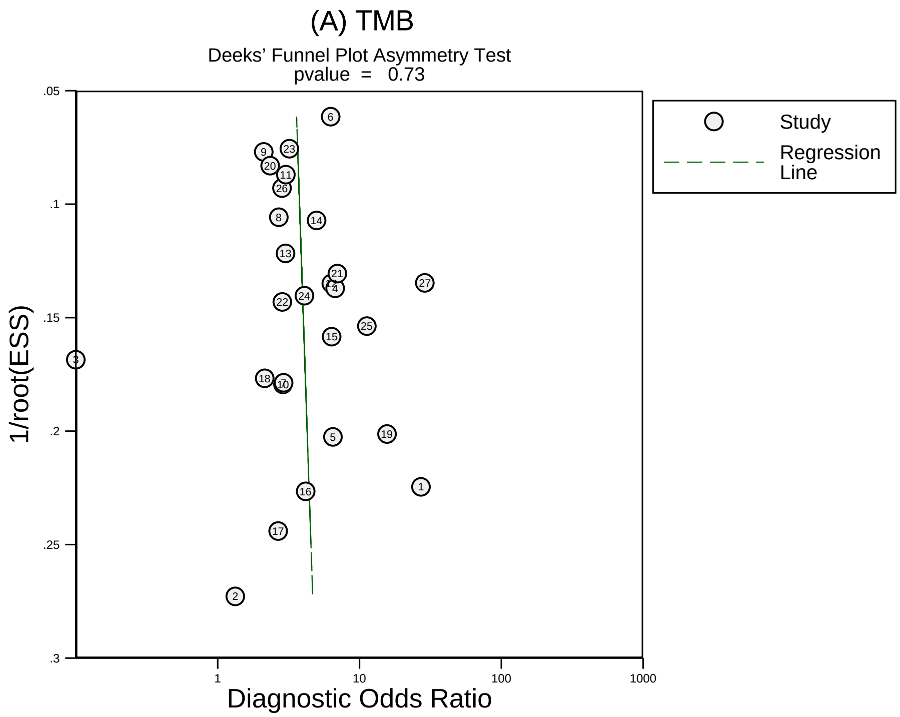

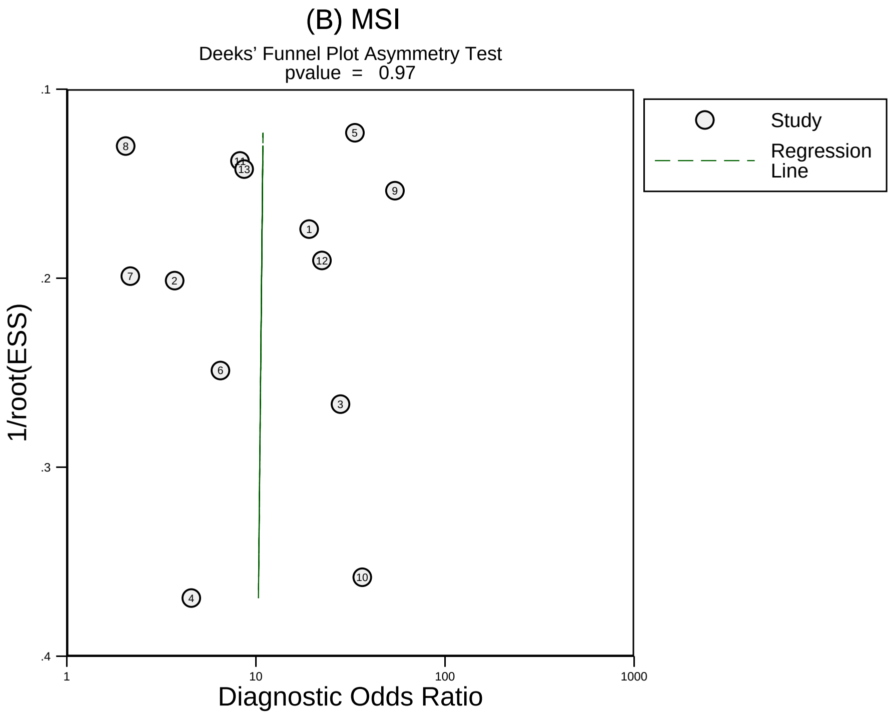

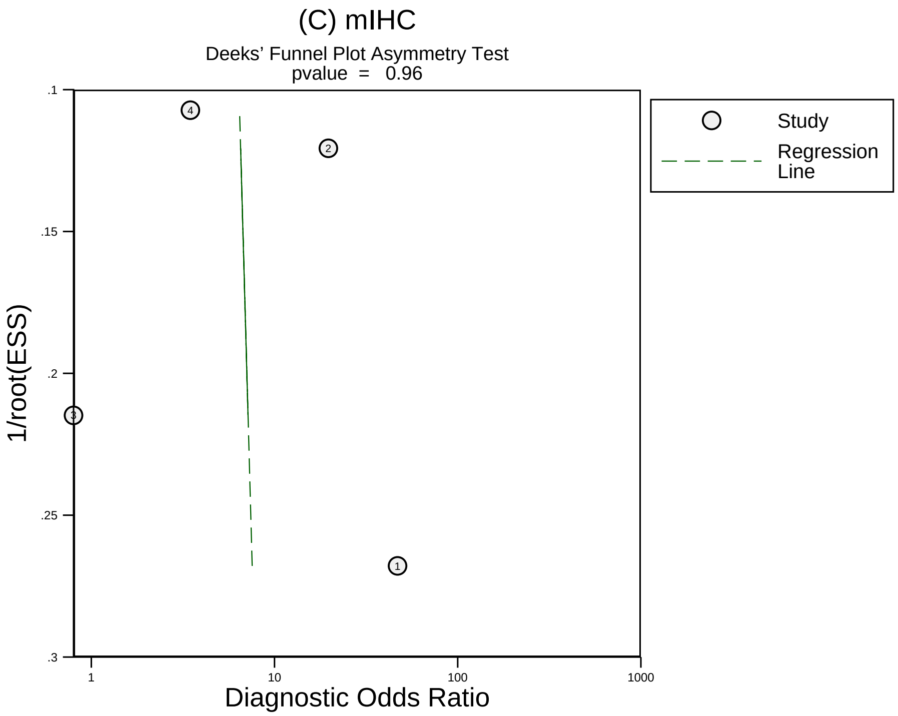

(C) mIHC
Deeks' Funnel Plot Asymmetry Test
pvalue = 0.96
Study
Regression Line
1/root(ESS)
Diagnostic Odds Ratio

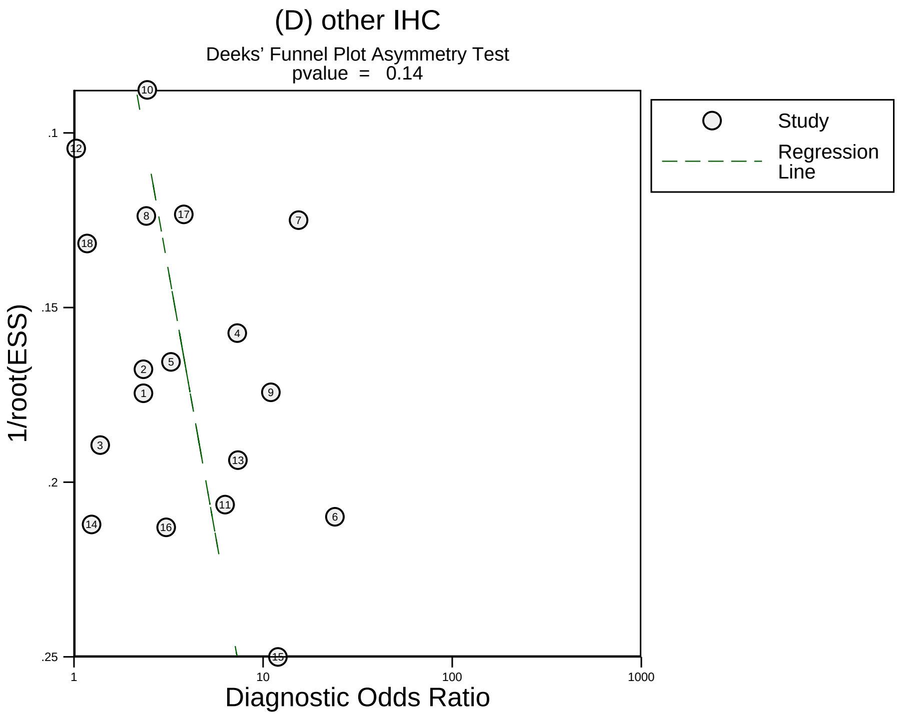

(D) other IHC
Deeks' Funnel Plot Asymmetry Test
pvalue = 0.14
Study
Regression Line
1/root(ESS)
Diagnostic Odds Ratio

(E) GEP

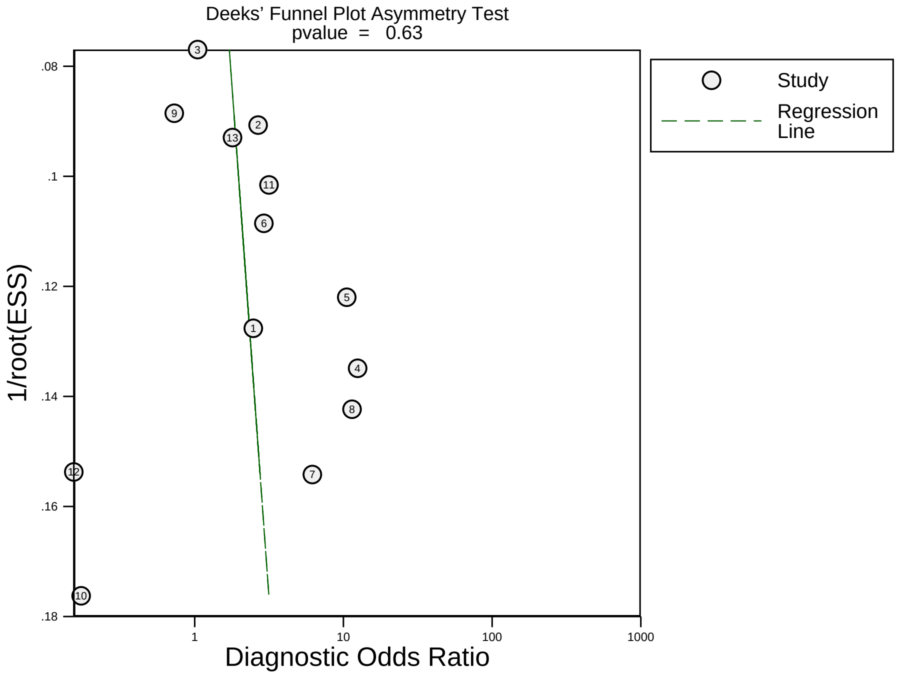

(F) combined assays

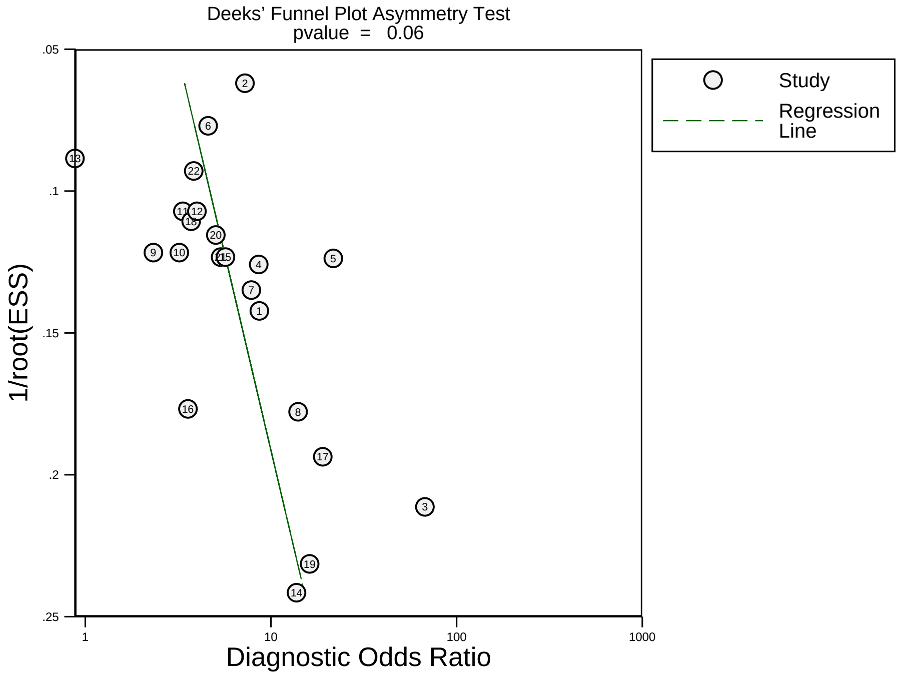

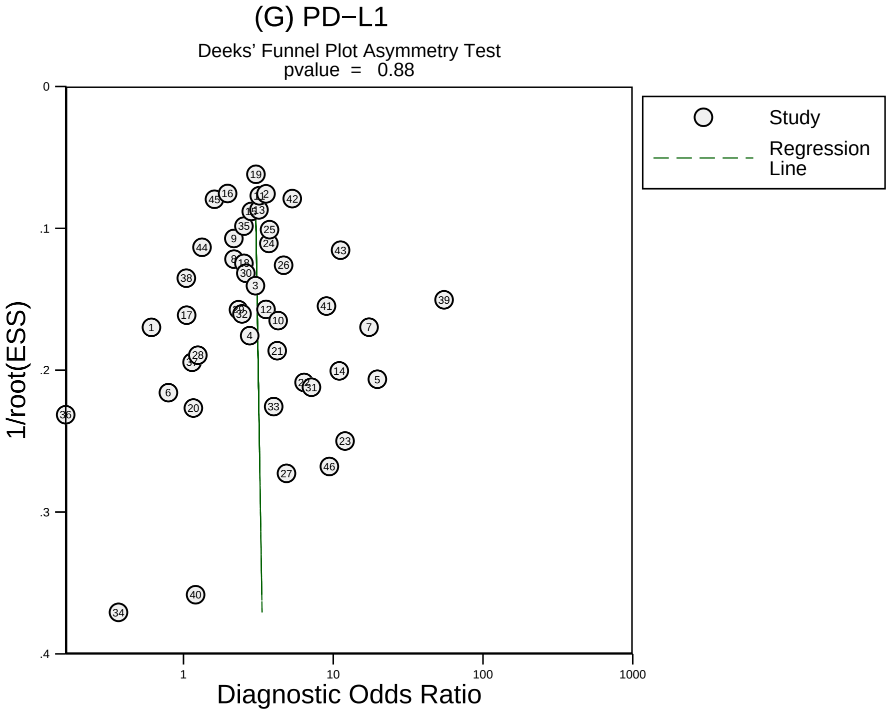

P-value presents publication bias.
Abbreviations: Programmed cell death ligand 1 immunohistochemistry (PD-L1 IHC); Tumor mutational burden (TMB); Gene expression profiling (GEP); Microsatellite instability (MSI); Multiplex immunohistochemistry /immunofluorescence (mIHC/IF); Other Immunohistochemistry and hematoxylin-eosin staining (other IHC&HE)
