# Supplementary material for: Comparison of different predictive biomarker testing assays for PD-1/PD-L1 checkpoint inhibitors response: a systematic review and network meta-analysis
Source: Front Immunol. 2023 Sep 26;14:1265202. doi: 10.3389/fimmu.2023.1265202 (PMC10562577; doi:10.3389/fimmu.2023.1265202)
Supplement: Supplementary file 4 [file Image_3.pdf]

Supplementary Figure 3. Sub-group Analysis of Combined assays by Meta-analysis

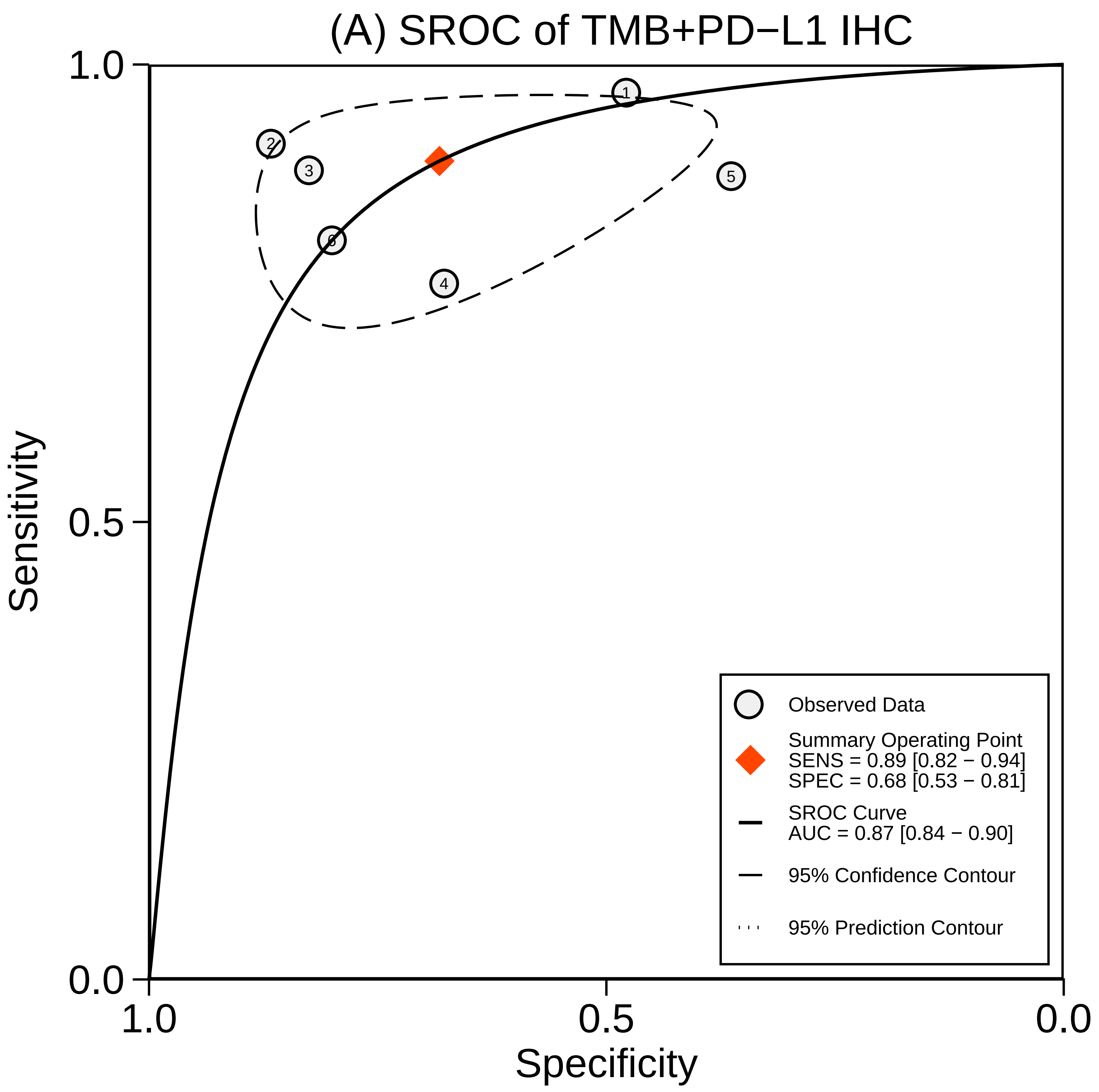

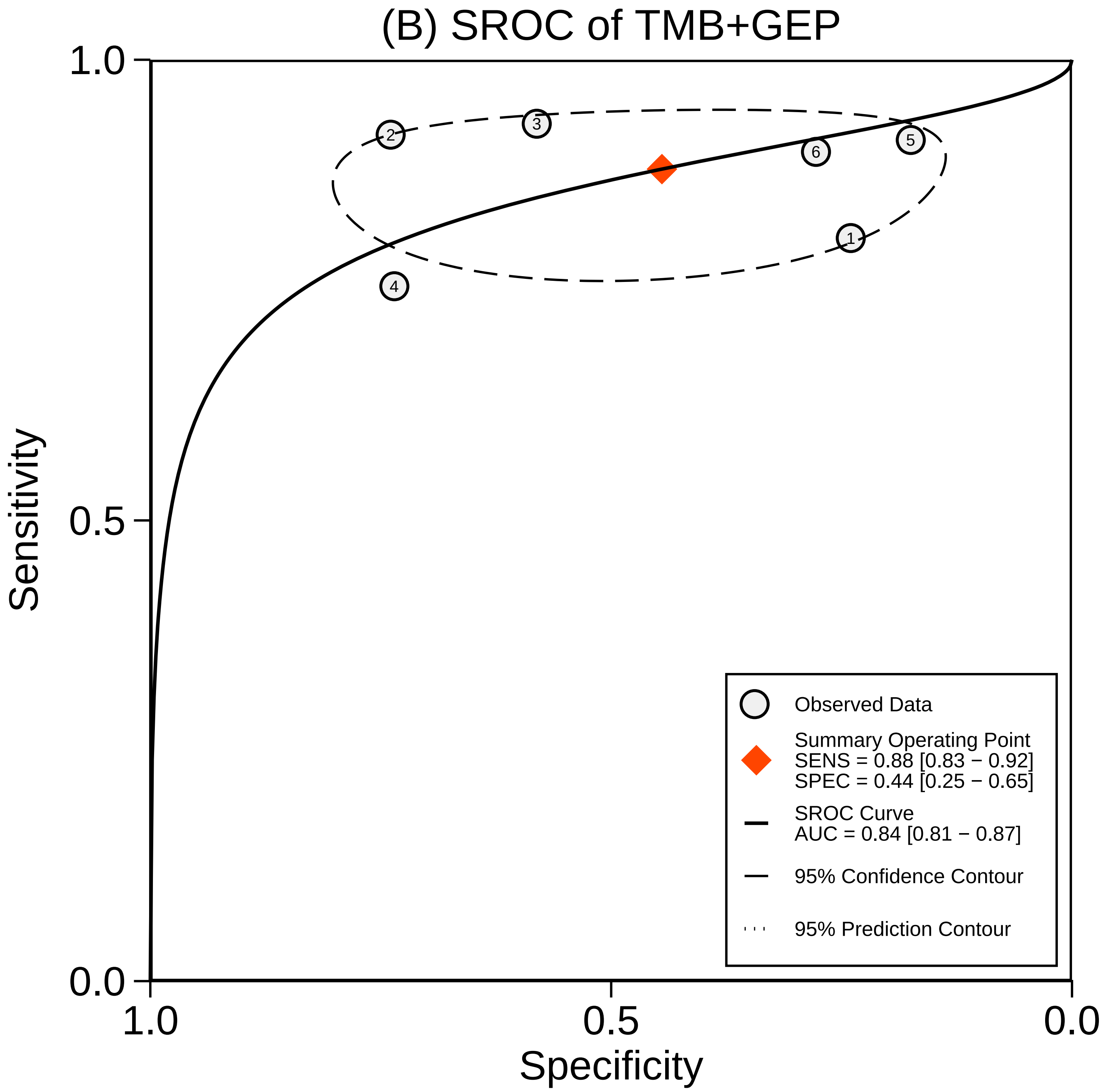
(B) SROC of TMB+GEP
Sensitivity
Specificity
1.0
0.5
0.0
1.0
0.5
0.0
1
2
3
4
5
6
Observed Data
Summary Operating Point
SENS = 0.88 [0.83 − 0.92]
SPEC = 0.44 [0.25 − 0.65]
SROC Curve
AUC = 0.84 [0.81 − 0.87]
95% Confidence Contour
95% Prediction Contour

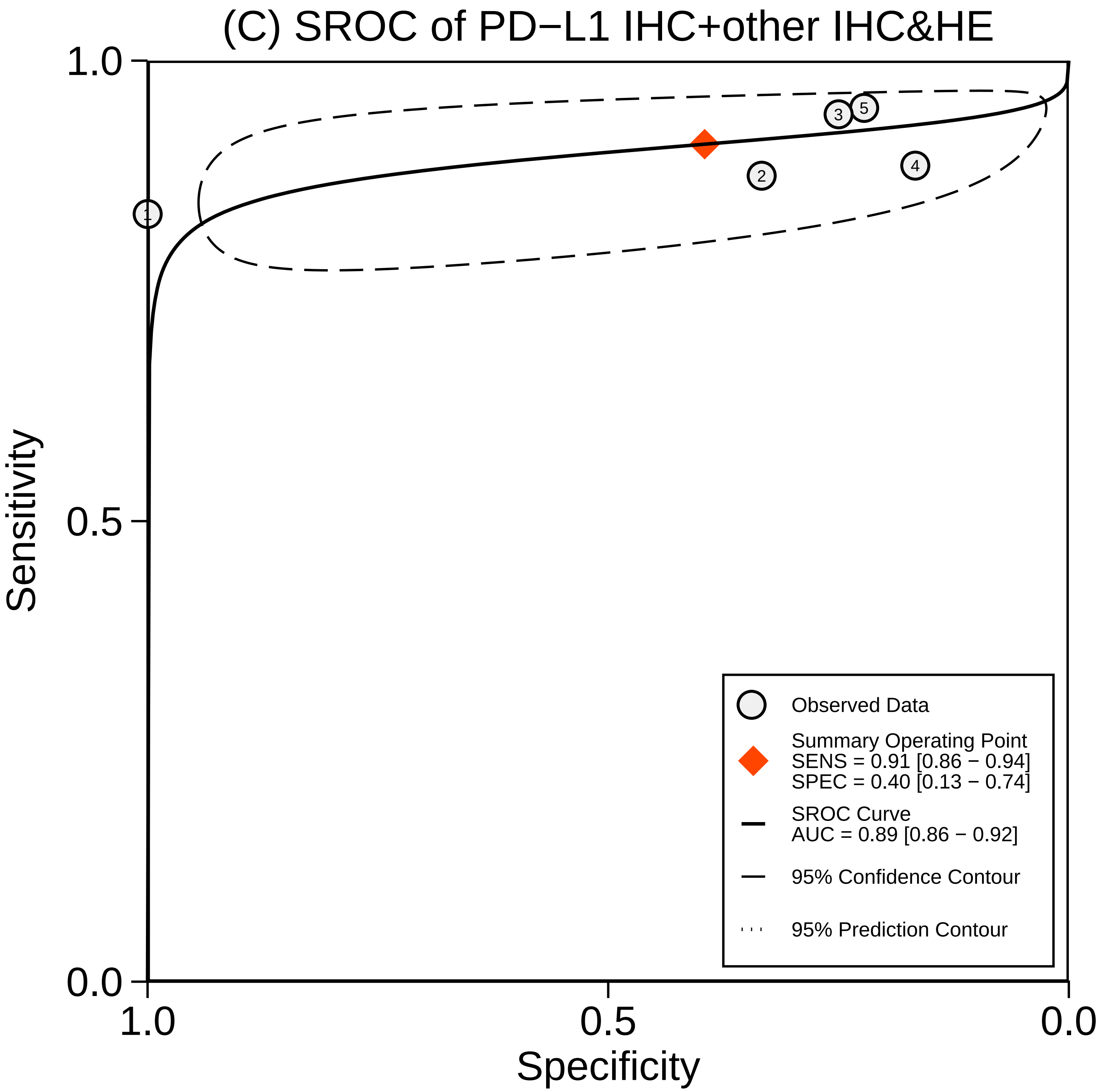

Abbreviations: Programmed cell death ligand 1 immunohistochemistry (PD-L1 IHC); Tumor mutational burden (TMB); Gene expression profiling (GEP); Other Immunohistochemistry and hematoxylin-eosin staining (other IHC&HE)
